# Supplementary material for: An open-source framework for large-scale, flexible evaluation of biomedical text mining systems
Source: J Biomed Discov Collab. 2008 Jan 29;3:1. doi: 10.1186/1747-5333-3-1 (PMC2276192; doi:10.1186/1747-5333-3-1)
Supplement: Additional file 1 — Evaluation platform source code. The additional file contains the software evaluation framework discussed in this paper. The most current release can be downloaded from SourceForge.net [1]. See the accompanying README file for instructions on installation and use. Also included in the distribution is a collection of UIMA wrappers for some commonly used BioNLP tools and annotated corpora. [file 1747-5333-3-1-S1.zip › bionlp_uima_repository-1.0.1/docs/api/src-html/edu/uchsc/ccp/uima/util/UIMA_Util.html]

```
001    /*
002     *
003     * The Original Code is UCHSC UIMA.
004     *
005     * The Initial Developer of the Original Code is University of Colorado.  
006     * Copyright (C) 2005.  All Rights Reserved.
007     *
008     * UCHSC Utilities was developed by the Center for Computational Pharmacology
009     * (http://compbio.uchsc.edu) at the University of Colorado Health 
010     *  Sciences Center School of Medicine with support from the National 
011     *  Library of Medicine.  
012     *
013     *
014     * Contributor(s):
015     *   William A. Baumgartner <william.baumgartner@uchsc.edu> (Original Author)
016     *   
017     * Created: Jan 2006
018     * Dependencies:
019     * TODO:
020     *          If any more Syntactic annotations are created, they should all be changed to use the UIMA ClassMention framework.. it is much more extensible.
021     * Changes:
022     *  05/04/06 - wab - added swapClassMentionInfo(), swapNonComplexSlotMentionInfo(), swapComplexSlotMentionInfo()
023     * 
024     */
025    package edu.uchsc.ccp.uima.util;
026    
027    import java.io.PrintStream;
028    import java.util.ArrayList;
029    import java.util.Arrays;
030    import java.util.HashMap;
031    import java.util.HashSet;
032    import java.util.Iterator;
033    import java.util.List;
034    import java.util.Set;
035    
036    import org.apache.uima.cas.CAS;
037    import org.apache.uima.cas.CASException;
038    import org.apache.uima.cas.ConstraintFactory;
039    import org.apache.uima.cas.FSIntConstraint;
040    import org.apache.uima.cas.FSMatchConstraint;
041    import org.apache.uima.cas.Feature;
042    import org.apache.uima.cas.FeaturePath;
043    import org.apache.uima.cas.FeatureStructure;
044    import org.apache.uima.cas.TypeSystem;
045    import org.apache.uima.jcas.JCas;
046    import org.apache.uima.jcas.cas.FSArray;
047    import org.apache.uima.jcas.cas.StringArray;
048    
049    import edu.uchsc.ccp.uima.annotation.CCPAnnotationSet;
050    import edu.uchsc.ccp.uima.annotation.CCPAnnotator;
051    import edu.uchsc.ccp.uima.annotation.CCPDocumentInformation;
052    import edu.uchsc.ccp.uima.annotation.CCPDocumentSection;
053    import edu.uchsc.ccp.uima.annotation.CCPSpan;
054    import edu.uchsc.ccp.uima.annotation.CCPTextAnnotation;
055    import edu.uchsc.ccp.uima.mention.CCPClassMention;
056    import edu.uchsc.ccp.uima.mention.CCPComplexSlotMention;
057    import edu.uchsc.ccp.uima.mention.CCPMention;
058    import edu.uchsc.ccp.uima.mention.CCPNonComplexSlotMention;
059    import edu.uchsc.ccp.uima.mention.CCPSlotMention;
060    import edu.uchsc.ccp.util.nlp.annotation.AnnotationSet;
061    import edu.uchsc.ccp.util.nlp.annotation.Annotator;
062    import edu.uchsc.ccp.util.nlp.annotation.InvalidSpanException;
063    import edu.uchsc.ccp.util.nlp.annotation.Span;
064    import edu.uchsc.ccp.util.nlp.annotation.TextAnnotation;
065    import edu.uchsc.ccp.util.nlp.document.DocumentSection;
066    import edu.uchsc.ccp.util.nlp.document.GenericDocument;
067    import edu.uchsc.ccp.util.nlp.mention.ClassMention;
068    import edu.uchsc.ccp.util.nlp.mention.ComplexSlotMention;
069    import edu.uchsc.ccp.util.nlp.mention.Mention;
070    import edu.uchsc.ccp.util.nlp.mention.SlotMention;
071    
072    /**
073     * This is a utility class for converting between UIMA annotations and TextAnnotations.
074     * 
075     * @author William A. Baumgartner, Jr.
076     * 
077     */
078    public class UIMA_Util {
079    
080        private static final boolean DEBUG = false;
081    
082        // /**
083        // * Convert a TextAnnotation to its UIMA corollary and add it to a JCAS
084        // *
085        // * @param jcas
086        // * @param ta
087        // */
088        // private static void textAnnotation2UIMA(JCas jcas, TextAnnotation ta) {
089        //
090        // /*
091        // * Is this a syntactic annotation, i.e. is it a Token, Phrase, or
092        // * Clause? If it is, then create the corresponding UIMA annotation. If
093        // * it is not, then create a UIMA semantic annotation.
094        // */
095        // edu.uchsc.ccp.util.nlp.mention.ClassMention cm = ta.getClassMention();
096        // if (cm.getMentionName().equalsIgnoreCase("token")) {
097        // // create a new TokenAnnotation
098        // TokenAnnotation tokenAnnotation = new TokenAnnotation(jcas);
099        //
100        // // extract the annotation information from the TextAnnotation and
101        // // fill the corresponding fields in the TokenAnnotation
102        // UIMA_Util.swapAnnotationInfo(ta, tokenAnnotation, jcas);
103        //
104        // // get POS
105        // SlotMention posSlot = cm.getSlotMentionByName("partOfSpeech");
106        // if (posSlot != null) {
107        // String posLabel = (String) posSlot.getSlotValues().get(0);
108        // tokenAnnotation.setPartOfSpeech(posLabel);
109        // } else {
110        // tokenAnnotation.setPartOfSpeech(null);
111        // }
112        //
113        // // get Lemma
114        // SlotMention lemmaSlot = cm.getSlotMentionByName("lemma");
115        // if (lemmaSlot != null) {
116        // String lemmaLabel = (String) lemmaSlot.getSlotValues().get(0);
117        // tokenAnnotation.setLemma(lemmaLabel);
118        // } else {
119        // tokenAnnotation.setLemma(null);
120        // }
121        //
122        // // get Stem
123        // SlotMention stemSlot = cm.getSlotMentionByName("stem");
124        // if (stemSlot != null) {
125        // String stemLabel = (String) stemSlot.getSlotValues().get(0);
126        // tokenAnnotation.setStem(stemLabel);
127        // } else {
128        // tokenAnnotation.setStem(null);
129        // }
130        //
131        // // add TokenAnnotation to CAS indexes
132        // tokenAnnotation.addToIndexes();
133        // } else if (cm.getMentionName().equalsIgnoreCase("phrase")) {
134        // PhraseAnnotation phraseAnnotation = new PhraseAnnotation(jcas);
135        //
136        // // extract the annotation information from the TextAnnotation and
137        // // fill the corresponding fields in the PhraseAnnotation
138        // UIMA_Util.swapAnnotationInfo(ta, phraseAnnotation, jcas);
139        //
140        // // get POS
141        // SlotMention phraseTypeSlot = cm.getSlotMentionByName("phraseType");
142        // if (phraseTypeSlot != null) {
143        // String phraseTypeLabel = (String) phraseTypeSlot.getSlotValues().get(0);
144        // phraseAnnotation.setPhraseType(phraseTypeLabel);
145        // } else {
146        // phraseAnnotation.setPhraseType(null);
147        // }
148        //
149        // // add PhraseAnnotation to CAS indexes
150        // phraseAnnotation.addToIndexes();
151        // } else if (cm.getMentionName().equalsIgnoreCase("clause")) {
152        // ClauseAnnotation clauseAnnotation = new ClauseAnnotation(jcas);
153        //
154        // // extract the annotation information from the TextAnnotation and
155        // // fill the corresponding fields in the PhraseAnnotation
156        // UIMA_Util.swapAnnotationInfo(ta, clauseAnnotation, jcas);
157        //
158        // // get POS
159        // SlotMention clauseTypeSlot = cm.getSlotMentionByName("clauseType");
160        // if (clauseTypeSlot != null) {
161        // String clauseTypeLabel = (String) clauseTypeSlot.getSlotValues().get(0);
162        // clauseAnnotation.setClauseType(clauseTypeLabel);
163        // } else {
164        // clauseAnnotation.setClauseType(null);
165        // }
166        //
167        // // add PhraseAnnotation to CAS indexes
168        // clauseAnnotation.addToIndexes();
169        // } else { // we are dealing with some kind of semantic annotation
170        // SemanticAnnotation semanticAnnotation = new SemanticAnnotation(jcas);
171        //
172        // // extract the annotation information from the TextAnnotation and
173        // // fill the corresponding fields in the SemanticAnnotation
174        // UIMA_Util.swapAnnotationInfo(ta, semanticAnnotation, jcas);
175        //
176        // // UIMA_Util.swapMentionInfo(ta, semanticAnnotation, jcas);
177        //
178        // // add the SemanticAnnotation to the CAS indexes
179        // semanticAnnotation.addToIndexes();
180        // }
181        //
182        // }
183    
184        // public static void swapClassMentionInfo(ClassMention fromCM, CCPClassMention toCCPClassMention, JCas jcas) {
185        // /* create a new CCPClassMention - this is somewhat redundant since we know this is already a Token, however it allows the annotation
186        // * to be used by tools which only use the more general semantic/syntactic annotations within the UIMA framework
187        // */
188        // // swap mention name
189        // toCCPClassMention.setMentionName(fromCM.getMentionName());
190        //
191        // // get the slot mentions to be put into the ccpClassMention
192        // Collection<SlotMention> slotMentions = fromCM.getSlotMentions();
193        // Collection<ComplexSlotMention> complexSlotMentions = fromCM.getComplexSlotMentions();
194        //        
195        // // init the array that will hold the ccp mentions
196        // FSArray ccpSlotMentionsArray = new FSArray(jcas, slotMentions.size() + complexSlotMentions.size());
197        // int mentionIndex = 0;
198        //        
199        // // for each slot mention, add it to the ccpSlotMentionsArray
200        // for (SlotMention sm : slotMentions) {
201        // CCPNonComplexSlotMention ccpNonComplexSlotMention = new CCPNonComplexSlotMention(jcas);
202        // swapSlotMentionInfo(sm, ccpNonComplexSlotMention, jcas);
203        // ccpSlotMentionsArray.set(mentionIndex++, ccpNonComplexSlotMention);
204        // }
205        //        
206        // // for each complex slot mention, add it to the ccpSlotMentionsArray
207        // for (ComplexSlotMention csm : complexSlotMentions) {
208        // // init a new CCPComplexSlotMention
209        // CCPComplexSlotMention ccpComplexSlotMention = new CCPComplexSlotMention(jcas);
210        // swapComplexSlotMentionInfo(csm, ccpComplexSlotMention, jcas);
211        // ccpSlotMentionsArray.set(mentionIndex++, ccpComplexSlotMention);
212        //            
213        // }
214        // toCCPClassMention.setSlotMentions(ccpSlotMentionsArray);
215        //        
216        // // set the text annotation references
217        // List<TextAnnotation> textAnnotations = fromCM.getTextAnnotations();
218        // for ()
219        // }
220        //    
221        // public static void swapSlotMentionInfo(SlotMention fromSM, CCPNonComplexSlotMention toCCPNonComplexSlotMention, JCas jcas) {
222        // toCCPNonComplexSlotMention.setMentionName(fromSM.getMentionName());
223        //
224        // // transfer the slot values
225        // List<Object> slotValues = fromSM.getSlotValues();
226        // StringArray slotValuesArray = new StringArray(jcas, slotValues.size());
227        // for (int i = 0; i < slotValues.size(); i++) {
228        // Object value = slotValues.get(i);
229        // if (value instanceof Integer) {
230        // slotValuesArray.set(i, ((Integer)value).toString());
231        // } else {
232        // // assume it's a String
233        // slotValuesArray.set(i, (String)value);
234        // }
235        // }
236        // toCCPNonComplexSlotMention.setSlotValues(slotValuesArray);
237        //        
238        // }
239        //    
240        // public static void swapComplexSlotMentionInfo(ComplexSlotMention fromCSM, CCPComplexSlotMention toCCPComplexSlotMention, JCas jcas) {
241        // toCCPComplexSlotMention.setMentionName(fromCSM.getMentionName());
242        //        
243        //        
244        // List<ClassMention> classMentions = fromCSM.getClassMentions();
245        // FSArray ccpClassMentionArray = new FSArray(jcas, classMentions.size());
246        // for (int i = 0; i < classMentions.size(); i++) {
247        // CCPClassMention ccpClassMention = new CCPClassMention(jcas);
248        // swapClassMentionInfo(classMentions.get(i), ccpClassMention, jcas);
249        // ccpClassMentionArray.set(i, ccpClassMention);
250        // }
251        // toCCPComplexSlotMention.setClassMentions(ccpClassMentionArray);
252        // }
253    
254        
255        public static String getDocumentID(JCas jcas) {
256            String documentID;
257          Iterator it = jcas.getJFSIndexRepository().getAnnotationIndex(CCPDocumentInformation.type).iterator();
258          if (it.hasNext()) { /* there will be at most one CCPDocumentInformation annotation */
259          CCPDocumentInformation docInfo = (CCPDocumentInformation) it.next();
260          documentID = docInfo.getDocumentID();
261          return documentID;
262          } else {
263              System.err.println("No document ID found, returning -1.");
264              return "-1";
265          }
266        }
267        
268        public static void swapDocumentInfo(JCas fromJcas, GenericDocument toGD) {
269            Iterator docInfoIter = fromJcas.getJFSIndexRepository().getAnnotationIndex(CCPDocumentInformation.type).iterator();
270            Iterator docSectionIter = fromJcas.getJFSIndexRepository().getAnnotationIndex(CCPDocumentSection.type).iterator();
271    
272            // print document information
273            String docID = "-1";
274            int docCollectionID = -1;
275            if (docInfoIter.hasNext()) {
276                CCPDocumentInformation docInfo = (CCPDocumentInformation) docInfoIter.next();
277                docID = docInfo.getDocumentID();
278                docCollectionID = docInfo.getDocumentCollectionID();
279                /* Get any secondary document IDs */
280                StringArray secondaryIDsArray = docInfo.getSecondaryDocumentIDs();
281                if (secondaryIDsArray != null) {
282                    for (int i = 0; i < secondaryIDsArray.size(); i++) {
283                        toGD.addSecondaryDocumentID(secondaryIDsArray.get(i));
284                    }
285                }
286            }
287    
288            toGD.setDocumentID(docID);
289            toGD.setDocumentCollectionID(docCollectionID);
290    
291            // add the document sections to the generic document
292            while (docSectionIter.hasNext()) {
293                CCPDocumentSection ccpDocSection = (CCPDocumentSection) docSectionIter.next();
294                DocumentSection docSection = new DocumentSection();
295                docSection.setDocumentSectionID(ccpDocSection.getSectionID());
296                docSection.setSectionStartIndex(ccpDocSection.getBegin());
297                docSection.setSectionEndIndex(ccpDocSection.getEnd());
298                toGD.addDocumentSection(docSection);
299            }
300    
301            // set the document text
302            toGD.setDocumentText(fromJcas.getDocumentText());
303    
304            // add the annotations to the Generic Document
305            UIMA_Util uimaUtil = new UIMA_Util();
306            List<TextAnnotation> annotations = uimaUtil.getTextAnnotationsFromJCas(fromJcas);
307            toGD.setAnnotations(annotations);
308    
309        }
310    
311        /**
312         * This method transfers general annotation info, i.e. span, annotator, etc. from a CCPTextAnnotation to a TextAnnotation
313         * 
314         * @param ccpAnnotation
315         * @param ta
316         */
317        public static void swapAnnotationInfo(CCPTextAnnotation fromUIMA, TextAnnotation toTA, JCas jcas) {
318    
319            // set the Annotation ID
320            toTA.setAnnotationID(fromUIMA.getAnnotationID());
321    
322            // set the Annotation Sets
323    
324            FSArray ccpAnnotationSets = fromUIMA.getAnnotationSets();
325            Set<AnnotationSet> annotationSets = new HashSet<AnnotationSet>();
326            if (ccpAnnotationSets != null) {
327                for (int i = 0; i < ccpAnnotationSets.size(); i++) {
328                    AnnotationSet annotationSet = new AnnotationSet(new Integer(-1), "", "");
329                    UIMA_Util.swapAnnotationSetInfo((CCPAnnotationSet) ccpAnnotationSets.get(i), annotationSet);
330                    annotationSets.add(annotationSet);
331                }
332            }
333            toTA.setAnnotationSets(annotationSets);
334    
335            // set the Annotator ID
336            CCPAnnotator ccpAnnotator = fromUIMA.getAnnotator();
337            Annotator annotator = new Annotator(new Integer(-1), "", "", "");
338            UIMA_Util.swapAnnotatorInfo(ccpAnnotator, annotator);
339            toTA.setAnnotator(annotator);
340    
341            // set the Span(s)
342            List<Span> spans = new ArrayList<Span>();
343            // FeatureStructure[] fsArray = fromUIMA.getSpans().toArray();
344            FSArray ccpSpans = fromUIMA.getSpans();
345            /* if there are no explicit spans in the CCPTextAnnotation, then we need to create a default span using the start/end indexes */
346            try {
347                if (ccpSpans.size() == 0) {
348                    Span span = new Span(fromUIMA.getStart(), fromUIMA.getEnd());
349                    spans.add(span);
350                } else {
351                    for (int i = 0; i < ccpSpans.size(); i++) {// FeatureStructure fs : fsArray) {
352                        CCPSpan ccpSpan = (CCPSpan) ccpSpans.get(i);
353                        Span span = new Span(ccpSpan.getSpanStart(), ccpSpan.getSpanEnd());
354                        spans.add(span);
355                    }
356                }
357            } catch (InvalidSpanException e) {
358                e.printStackTrace();
359            }
360    
361            toTA.setSpans(spans);
362    
363            // set the DocumentSection ID
364            toTA.setDocumentSectionID(fromUIMA.getDocumentSectionID());
365    
366            // set the Document ID and Document Collection ID
367            Iterator docInfoIter = jcas.getJFSIndexRepository().getAnnotationIndex(CCPDocumentInformation.type).iterator();
368    
369            // get the document ID and document collection ID
370            String docID = "-1";
371            int docCollectionID = -1;
372            if (docInfoIter.hasNext()) {
373                CCPDocumentInformation docInfo = (CCPDocumentInformation) docInfoIter.next();
374                docID = docInfo.getDocumentID();
375                docCollectionID = docInfo.getDocumentCollectionID();
376            } else {
377                System.err.println("CAS is missing CCPDocumentInformation. Document ID and DocumentCollection ID have been set to -1.");
378            }
379            toTA.setDocumentID(docID);
380            toTA.setDocumentCollectionID(docCollectionID);
381    
382            // set the Covered Text
383            try {
384            toTA.setCoveredText(fromUIMA.getCoveredText());
385            } catch (Exception e) {
386                System.err.println("EXCEPTION: " + jcas.getDocumentText());
387            }
388        }
389    
390        /**
391         * Swap from UIMA Annotation to another UIMA Annotation
392         * 
393         * @param fromUIMA
394         * @param toUIMA
395         * @param jcas
396         */
397        public static void swapAnnotationInfo(CCPTextAnnotation fromUIMA, CCPTextAnnotation toUIMA) throws CASException {
398            // set the Annotation ID
399            toUIMA.setAnnotationID(fromUIMA.getAnnotationID());
400    
401            // set the Annotation Sets
402            toUIMA.setAnnotationSets(fromUIMA.getAnnotationSets());
403    
404            // set the Annotator ID
405            toUIMA.setAnnotator(fromUIMA.getAnnotator());
406    
407            // set the Span(s)
408            toUIMA.setSpans(fromUIMA.getSpans());
409    
410            /* set the number of spans */
411            toUIMA.setNumberOfSpans(fromUIMA.getNumberOfSpans());
412    
413            // set the default Begin and End fields
414            toUIMA.setBegin(fromUIMA.getBegin());
415            toUIMA.setEnd(fromUIMA.getEnd());
416    
417            // set the DocumentSection ID
418            toUIMA.setDocumentSectionID(fromUIMA.getDocumentSectionID());
419    
420            /* swap class mentions */
421            CCPClassMention ccpCM = new CCPClassMention(fromUIMA.getCAS().getJCas());
422            UIMA_Util.swapClassMentionInfo(fromUIMA.getClassMention(), ccpCM);
423            toUIMA.setClassMention(ccpCM);
424        }
425    
426        public static void swapClassMentionInfo(CCPClassMention fromCM, CCPClassMention toCM) throws CASException {
427            toCM.setMentionName(fromCM.getMentionName());
428            JCas jcas = fromCM.getCAS().getJCas();
429            FSArray fromSlotMentions = fromCM.getSlotMentions();
430            if (fromSlotMentions != null) {
431                FSArray toSlotMentions = new FSArray(jcas, fromSlotMentions.size());
432                for (int i = 0; i < fromSlotMentions.size(); i++) {
433                    if (fromSlotMentions.get(i) instanceof CCPNonComplexSlotMention) {
434                        CCPNonComplexSlotMention toSM = new CCPNonComplexSlotMention(jcas);
435                        UIMA_Util.swapNonComplexSlotMentionInfo((CCPNonComplexSlotMention) fromSlotMentions.get(i), toSM);
436                        toSlotMentions.set(i, toSM);
437                    } else if (fromSlotMentions.get(i) instanceof CCPComplexSlotMention) {
438                        CCPComplexSlotMention toSM = new CCPComplexSlotMention(jcas);
439                        UIMA_Util.swapComplexSlotMentionInfo((CCPComplexSlotMention) fromSlotMentions.get(i), toSM);
440                        toSlotMentions.set(i, toSM);
441                    } else {
442                        System.err.println("Expecting CCPNonComplexSlotMention of CCPComplexSlotMention but got: "
443                                + fromSlotMentions.get(i).getClass().getName());
444                    }
445                }
446                toCM.setSlotMentions(toSlotMentions);
447            }
448    
449    //        try {
450            FSArray fromTextAnnotations = fromCM.getCcpTextAnnotations();
451            if (fromTextAnnotations != null) {
452                toCM.setCcpTextAnnotations(fromTextAnnotations);
453            }
454    //        } catch (CASRuntimeException casre) {
455    //              // do nothing. Before UIMA 2.0, this used to return null.. now it throws an exception.
456    //        }
457    
458        }
459    
460        public static void swapNonComplexSlotMentionInfo(CCPNonComplexSlotMention fromSM, CCPNonComplexSlotMention toSM) throws CASException {
461            toSM.setMentionName(fromSM.getMentionName());
462            JCas jcas = fromSM.getCAS().getJCas();
463            StringArray fromSlotValues = fromSM.getSlotValues();
464            if (fromSlotValues != null) {
465                StringArray toSlotValues = new StringArray(jcas, fromSlotValues.size());
466                for (int i = 0; i < fromSlotValues.size(); i++) {
467                    toSlotValues.set(i, fromSlotValues.get(i));
468                }
469                toSM.setSlotValues(toSlotValues);
470            }
471    
472        }
473    
474        public static void swapComplexSlotMentionInfo(CCPComplexSlotMention fromSM, CCPComplexSlotMention toSM) throws CASException {
475            toSM.setMentionName(fromSM.getMentionName());
476            JCas jcas = fromSM.getCAS().getJCas();
477            FSArray fromClassMentions = fromSM.getClassMentions();
478            if (fromClassMentions != null) {
479                FSArray toClassMentions = new FSArray(jcas, fromClassMentions.size());
480                for (int i = 0; i < fromClassMentions.size(); i++) {
481                    CCPClassMention toCM = new CCPClassMention(jcas);
482                    UIMA_Util.swapClassMentionInfo((CCPClassMention) fromClassMentions.get(i), toCM);
483                    toClassMentions.set(i, toCM);
484                }
485                toSM.setClassMentions(toClassMentions);
486            }
487        }
488    
489        /**
490         * This method transfers general annotation info, i.e. span, annotator, etc. from a TextAnnotation to a CCPTextAnnotation.
491         * 
492         * @param ta
493         * @param ccpAnnotation
494         */
495        public static void swapAnnotationInfo(TextAnnotation fromTA, CCPTextAnnotation toUIMA, JCas jcas) {
496            // System.err.println("DEBUG -- UIMA_Util: creating UIMA annotationID:" + fromTA.getAnnotationID());
497    
498            // set the Annotation ID
499            toUIMA.setAnnotationID(fromTA.getAnnotationID());
500    
501            // validate the Annotation Set ID(s) against the known annotation sets
502    
503            // set the Annotation Sets
504            Set<AnnotationSet> annotationSets = fromTA.getAnnotationSets();
505            FSArray ccpAnnotationSets = new FSArray(jcas, annotationSets.size());
506            int index = 0;
507            for (AnnotationSet aSet : annotationSets) {
508                // for (int i = 0; i < annotationSets.size(); i++) {
509                CCPAnnotationSet ccpAnnotationSet = new CCPAnnotationSet(jcas);
510                UIMA_Util.swapAnnotationSetInfo(aSet, ccpAnnotationSet);
511                ccpAnnotationSets.set(index++, ccpAnnotationSet);
512            }
513            toUIMA.setAnnotationSets(ccpAnnotationSets);
514            CCPAnnotator ccpAnnotator = new CCPAnnotator(jcas);
515            UIMA_Util.swapAnnotatorInfo(fromTA.getAnnotator(), ccpAnnotator);
516            toUIMA.setAnnotator(ccpAnnotator);
517    
518            /*
519             * set the Span(s) A CCPSpan object is created for each Span object associated with the TextAnnotation. Also, the default Begin and End fields
520             * for the UIMA annotation are set to the min and max indexes of all Span objects.
521             */
522            int minSpanIndex = 999999999;
523            int maxSpanIndex = -1;
524            ArrayList<Span> spans = (ArrayList<Span>) fromTA.getSpans();
525    
526            // System.err.println("DEBUG -- UIMA_Util: # spans:" + spans.size());
527            toUIMA.setNumberOfSpans(spans.size());
528            FSArray supplementarySpans = new FSArray(jcas, spans.size());
529            for (int i = 0; i < spans.size(); i++) {
530                Span span = spans.get(i);
531                if (minSpanIndex > span.getSpanStart()) {
532                    minSpanIndex = span.getSpanStart();
533                }
534                if (maxSpanIndex < span.getSpanEnd()) {
535                    maxSpanIndex = span.getSpanEnd();
536                }
537                // // by default set the Begin and End values of the UIMA annotation
538                // // equal to those of the first Span
539                // if (i == 0) {
540                // toUIMA.setBegin(span.getSpanStart());
541                // toUIMA.setEnd(span.getSpanEnd());
542                // }
543                // for each TextAnnotation Span, add a UIMA Span to the
544                // CCPTextAnnotation
545    
546                CCPSpan uimaSpan = new CCPSpan(jcas);
547                uimaSpan.setSpanStart(span.getSpanStart());
548                uimaSpan.setSpanEnd(span.getSpanEnd());
549                supplementarySpans.set(i, uimaSpan);
550            }
551            toUIMA.setSpans(supplementarySpans);
552            toUIMA.setBegin(minSpanIndex);
553            toUIMA.setEnd(maxSpanIndex);
554    
555            // set the DocumentSection ID
556            toUIMA.setDocumentSectionID(fromTA.getDocumentSectionID());
557    
558            // System.err.println("DEBUG -- UIMA_Util: span begin: " + toUIMA.getBegin() + " span end: " + toUIMA.getEnd());
559    
560        }
561    
562        public static void swapAnnotatorInfo(CCPAnnotator ccpAnnotator, Annotator annotator) {
563            if (ccpAnnotator != null) {
564                annotator.setAnnotatorID(new Integer(ccpAnnotator.getAnnotatorID()));
565                annotator.setFirstName(ccpAnnotator.getFirstName());
566                annotator.setLastName(ccpAnnotator.getLastName());
567                annotator.setAffiliation(ccpAnnotator.getAffiliation());
568            }
569        }
570    
571        public static void swapAnnotatorInfo(Annotator annotator, CCPAnnotator ccpAnnotator) {
572            if (annotator != null) {
573                ccpAnnotator.setAnnotatorID(annotator.getAnnotatorID().intValue());
574                ccpAnnotator.setFirstName(annotator.getFirstName());
575                ccpAnnotator.setLastName(annotator.getLastName());
576                ccpAnnotator.setAffiliation(annotator.getAffiliation());
577            }
578        }
579    
580        public static void swapAnnotationSetInfo(CCPAnnotationSet ccpAnnotationSet, AnnotationSet annotationSet) {
581            if (ccpAnnotationSet != null) {
582                annotationSet.setAnnotationSetID(new Integer(ccpAnnotationSet.getAnnotationSetID()));
583                annotationSet.setAnnotationSetName(ccpAnnotationSet.getAnnotationSetName());
584                annotationSet.setAnnotationSetDescription(ccpAnnotationSet.getAnnotationSetDescription());
585            }
586        }
587    
588        public static void swapAnnotationSetInfo(AnnotationSet annotationSet, CCPAnnotationSet ccpAnnotationSet) {
589            if (annotationSet != null) {
590                ccpAnnotationSet.setAnnotationSetID(annotationSet.getAnnotationSetID().intValue());
591                ccpAnnotationSet.setAnnotationSetName(annotationSet.getAnnotationSetName());
592                ccpAnnotationSet.setAnnotationSetDescription(annotationSet.getAnnotationSetDescription());
593            }
594        }
595    
596        // /**
597        // * This method transfers the mention structure of a TextAnnotation to a
598        // UIMA
599        // * CCPTextAnnotation
600        // *
601        // * @param fromTA
602        // * @param toUIMA
603        // * @param jcas
604        // */
605        // public static void swapMentionInfo(TextAnnotation fromTA,
606        // SemanticAnnotation toUIMA, JCas jcas) {
607        // // edu.uchsc.ccp.util.nlp.annotation.ClassMention cm =
608        // // fromTA.getClassMention();
609        // //
610        // // edu.uchsc.ccp.uima.annotation.semantic.mention.ClassMention uimaCM =
611        // // new
612        // // edu.uchsc.ccp.uima.annotation.semantic.mention.ClassMention(jcas);
613        // //
614        // //
615        // //
616        // //
617        // // toUIMA.setClassMention(uimaCM);
618        // }
619        //
620        // public static void swapMentionInfo(
621        // edu.uchsc.ccp.util.nlp.mention.Mention fromMention,
622        // edu.uchsc.ccp.uima.annotation.semantic.mention.CCPMention toUIMAMention,
623        // JCas jcas) {
624        //
625        // // if edu.uchsc.ccp.util.nlp.annotation.ClassMention cm =
626        // // fromTA.getClassMention();
627        // //
628        // //
629        // //
630        // // edu.uchsc.ccp.uima.annotation.semantic.mention.ClassMention uimaCM =
631        // // new
632        // // edu.uchsc.ccp.uima.annotation.semantic.mention.ClassMention(jcas);
633        // //
634        // //
635        // //
636        // //
637        // // toUIMA.setClassMention(uimaCM);
638        // }
639        //
640        //  
641        //
642        //  
643        // /**
644        // * This method transfers the mention structure of a UIMA CCPTextAnnotation
645        // * to a TextAnnotation
646        // *
647        // * @param fromTA
648        // * @param toUIMA
649        // * @param jcas
650        // */
651        // public static void swapMentionInfo(SemanticAnnotation fromUIMA,
652        // TextAnnotation toTA, JCas jcas) {
653        // edu.uchsc.ccp.uima.annotation.semantic.mention.ClassMention uimaCM =
654        // fromUIMA
655        // .getClassMention();
656        // edu.uchsc.ccp.util.nlp.mention.ClassMention cm = new
657        // edu.uchsc.ccp.util.nlp.mention.ClassMention();
658        //  
659        // toTA.setClassMention(cm);
660        //  
661        // }
662    
663        public List<TextAnnotation> getTextAnnotationsFromJCas(JCas jcas) {
664            List<TextAnnotation> textAnnotations = new ArrayList<TextAnnotation>();
665    
666            Iterator uimaAnnotationIter = jcas.getJFSIndexRepository().getAnnotationIndex(CCPTextAnnotation.type).iterator();
667    
668            HashMap<String, TextAnnotation> alreadyExtractedAnnotations = new HashMap<String, TextAnnotation>();
669            HashMap<String, ClassMention> alreadyExtractedMentions = new HashMap<String, ClassMention>();
670    
671            // for each knowtator annotation, create a corresponding TextAnnotation
672            while (uimaAnnotationIter.hasNext()) {
673                CCPTextAnnotation ccpTA = (CCPTextAnnotation) uimaAnnotationIter.next();
674                TextAnnotation ta = extractTextAnnotation(ccpTA, jcas, alreadyExtractedAnnotations, alreadyExtractedMentions, null);
675                textAnnotations.add(ta);
676            }
677    
678            if (DEBUG) {
679                System.out.println("#############################################");
680                System.out.println("# of alreadyExtractedAnnotations = " + alreadyExtractedAnnotations.size());
681                Iterator keyIter = alreadyExtractedAnnotations.keySet().iterator();
682                while (keyIter.hasNext()) {
683                    System.out.println("annotation key: " + keyIter.next());
684                }
685                System.out.println("# of alreadyExtractedMentions = " + alreadyExtractedMentions.size());
686                keyIter = alreadyExtractedMentions.keySet().iterator();
687                while (keyIter.hasNext()) {
688                    System.out.println("mention key: " + keyIter.next());
689                }
690                System.out.println("#############################################");
691            }
692            return textAnnotations;
693        }
694    
695        // private TextAnnotation extractTextAnnotation(CCPTextAnnotation ccpTA,
696        // JCas jcas,
697        // HashMap<String, TextAnnotation> alreadyExtractedAnnotations,
698        // HashMap<String, ClassMention> alreadyExtractedMentions,
699        // ClassMention classMention, AnnotationSetValidator annotationSetValidator,
700        // AnnotatorValidator annotatorValidator) {
701        //
702        // TextAnnotation ta = new TextAnnotation();
703        // swapAnnotationInfo(ccpTA, ta, jcas);
704        //
705        // if (ccpTA instanceof TokenAnnotation) {
706        // processUIMATokenAnnotation(ccpTA, ta);
707        // } else if (ccpTA instanceof PhraseAnnotation) {
708        // processUIMAPhraseAnnotation(ccpTA, ta);
709        // } else if (ccpTA instanceof ClauseAnnotation) {
710        // processUIMAClauseAnnotation(ccpTA, ta);
711        // } else if (ccpTA instanceof SemanticAnnotation) {
712        // processUIMASemanticAnnotation(ccpTA, ta, jcas,
713        // alreadyExtractedAnnotations, alreadyExtractedMentions, classMention,
714        // annotationSetValidator, annotatorValidator);
715        // } else {
716        // System.out.println("Unhandled annotation type trying to be converted to
717        // TextAnnotation...");
718        // }
719        //
720        // return ta;
721        // }
722    
723        // private void processUIMATokenAnnotation(CCPTextAnnotation ccpTA,
724        // TextAnnotation ta) {
725        // TokenAnnotation tokenAnnotation = (TokenAnnotation) ccpTA;
726        //
727        // // create a ClassMention with partOfSpeech, lemma, and stem slots
728        // edu.uchsc.ccp.util.nlp.mention.ClassMention cm = new
729        // edu.uchsc.ccp.util.nlp.mention.ClassMention();
730        // cm.setMentionName("token");
731        //
732        // if (tokenAnnotation.getPartOfSpeech() != null) {
733        // edu.uchsc.ccp.util.nlp.mention.SlotMention posSlot = new
734        // edu.uchsc.ccp.util.nlp.mention.SlotMention();
735        // posSlot.setMentionName("partOfSpeech");
736        // posSlot.addSlotValue(tokenAnnotation.getPartOfSpeech());
737        // cm.addSlotMention(posSlot);
738        // }
739        //
740        // if (tokenAnnotation.getLemma() != null) {
741        // edu.uchsc.ccp.util.nlp.mention.SlotMention lemmaSlot = new
742        // edu.uchsc.ccp.util.nlp.mention.SlotMention();
743        // lemmaSlot.setMentionName("lemma");
744        // lemmaSlot.addSlotValue(tokenAnnotation.getLemma());
745        // cm.addSlotMention(lemmaSlot);
746        // }
747        // if (tokenAnnotation.getStem() != null) {
748        // edu.uchsc.ccp.util.nlp.mention.SlotMention stemSlot = new
749        // edu.uchsc.ccp.util.nlp.mention.SlotMention();
750        // stemSlot.setMentionName("stem");
751        // stemSlot.addSlotValue(tokenAnnotation.getStem());
752        // cm.addSlotMention(stemSlot);
753        // }
754        // ta.setClassMention(cm);
755        // }
756        //
757        // private void processUIMAPhraseAnnotation(CCPTextAnnotation ccpTA,
758        // TextAnnotation ta) {
759        // PhraseAnnotation phraseAnnotation = (PhraseAnnotation) ccpTA;
760        //
761        // // create a ClassMention with phraseType slot
762        // edu.uchsc.ccp.util.nlp.mention.ClassMention cm = new
763        // edu.uchsc.ccp.util.nlp.mention.ClassMention();
764        // cm.setMentionName("phrase");
765        //
766        // if (phraseAnnotation.getPhraseType() != null) {
767        // edu.uchsc.ccp.util.nlp.mention.SlotMention phraseTypeSlot = new
768        // edu.uchsc.ccp.util.nlp.mention.SlotMention();
769        // phraseTypeSlot.setMentionName("phraseType");
770        // phraseTypeSlot.addSlotValue(phraseAnnotation.getPhraseType());
771        // cm.addSlotMention(phraseTypeSlot);
772        // }
773        //        
774        // ta.setClassMention(cm);
775        // }
776        //
777        // private void processUIMAClauseAnnotation(CCPTextAnnotation ccpTA,
778        // TextAnnotation ta) {
779        // ClauseAnnotation clauseAnnotation = (ClauseAnnotation) ccpTA;
780        //
781        // // create a ClassMention with clauseType slot
782        // edu.uchsc.ccp.util.nlp.mention.ClassMention cm = new
783        // edu.uchsc.ccp.util.nlp.mention.ClassMention();
784        // cm.setMentionName("clause");
785        //
786        // if (clauseAnnotation.getClauseType() != null) {
787        // edu.uchsc.ccp.util.nlp.mention.SlotMention clauseTypeSlot = new
788        // edu.uchsc.ccp.util.nlp.mention.SlotMention();
789        // clauseTypeSlot.setMentionName("clauseType");
790        // clauseTypeSlot.addSlotValue(clauseAnnotation.getClauseType());
791        // cm.addSlotMention(clauseTypeSlot);
792        // }
793        // ta.setClassMention(cm);
794        // }
795    
796        // private void processUIMASemanticAnnotation(CCPTextAnnotation ccpTA,
797        // TextAnnotation ta, JCas jcas,
798        // HashMap<String, TextAnnotation> alreadyExtractedAnnotations,
799        // HashMap<String, ClassMention> alreadyExtractedMentions,
800        // ClassMention classMention, AnnotationSetValidator annotationSetValidator,
801        // AnnotatorValidator annotatorValidator) {
802    
803        private TextAnnotation extractTextAnnotation(CCPTextAnnotation ccpTA, JCas jcas,
804                HashMap<String, TextAnnotation> alreadyExtractedAnnotations, HashMap<String, ClassMention> alreadyExtractedMentions,
805                ClassMention classMention) {
806    
807            TextAnnotation ta = new TextAnnotation();
808            swapAnnotationInfo(ccpTA, ta, jcas);
809    
810            // SemanticAnnotation semanticAnnotation = (SemanticAnnotation) ccpTA;
811    
812            // if
813            // (!alreadyExtractedAnnotations.containsKey(getSemanticAnnotationHashKey(semanticAnnotation)))
814            // {
815            if (!alreadyExtractedAnnotations.containsKey(getCCPTextAnnotationHashKey(ccpTA))) {
816    
817                // if mention is null, then create a new Util ClassMention from the
818                // associated Knowtator mention, else, use the inputted mention.
819                // this prevents infinite loops from occurring when creating an
820                // annotation from a ClassMention that was found in a
821                // complexSlotMention.
822                if (classMention == null) {
823                    // create associated Util ClassMention
824                    // CCPClassMention ccpClassMention =
825                    // semanticAnnotation.getClassMention();
826                    CCPClassMention ccpClassMention = ccpTA.getClassMention();
827                    classMention = (ClassMention) extractUtilMention(ccpClassMention, jcas, alreadyExtractedAnnotations,
828                            alreadyExtractedMentions);
829                }
830    
831                // associate this TextAnnotation with the classMention
832                classMention.addTextAnnotation(ta);
833    
834                // set the ClassMention for the TextAnnotation
835                ta.setClassMention(classMention);
836    
837                // add the TextAnnotation to alreadyExtractedAnnotations if it is
838                // not already there
839                // Here, the check is just a safeguard. The TextAnnotation should
840                // always be added to the Hash here.
841                if (!alreadyExtractedAnnotations.containsKey(ta.getSingleLineRepresentation(true, true))) {
842                    alreadyExtractedAnnotations.put(ta.getSingleLineRepresentation(true, true), ta);
843                }
844            } else {
845                // ta =
846                // alreadyExtractedAnnotations.get(getSemanticAnnotationHashKey(semanticAnnotation));
847                ta = alreadyExtractedAnnotations.get(getCCPTextAnnotationHashKey(ccpTA));
848            }
849            // UIMA_Util.swapMentionInfo(semanticAnnotation, ta, jcas);
850    
851            return ta;
852    
853        }
854    
855        private Mention extractUtilMention(CCPMention ccpMention, JCas jcas, HashMap<String, TextAnnotation> alreadyExtractedAnnotations,
856                HashMap<String, ClassMention> alreadyExtractedMentions) {
857            Mention returnMention;
858    
859            if (ccpMention instanceof CCPClassMention) {
860                returnMention = processCCPClassMention(ccpMention, jcas, alreadyExtractedAnnotations, alreadyExtractedMentions);
861            } else if (ccpMention instanceof CCPComplexSlotMention) {
862                returnMention = processCCPComplexSlotMention(ccpMention, jcas, alreadyExtractedAnnotations, alreadyExtractedMentions);
863            } else if (ccpMention instanceof CCPNonComplexSlotMention) {
864                returnMention = processCCPSlotMention(ccpMention, jcas, alreadyExtractedAnnotations, alreadyExtractedMentions);
865            } else {
866                if (ccpMention == null) {
867                    System.err
868                            .println("A null mention has been found. Please make sure each CCPTextAnnotation is associated with a CCPClassMention.");
869                } else {
870                    System.err.println("The mention you are trying to create is an instance of: " + ccpMention.getClass().getName());
871                    System.err.println("Currently, only ClassMentions, ComplexSlotMentions, and SlotMentions can be added.");
872                }
873                returnMention = null;
874            }
875            return returnMention;
876        }
877    
878        private ClassMention processCCPClassMention(CCPMention ccpMention, JCas jcas,
879                HashMap<String, TextAnnotation> alreadyExtractedAnnotations, HashMap<String, ClassMention> alreadyExtractedMentions) {
880    
881            // create a new Util ClassMention
882            CCPClassMention ccpClassMention = (CCPClassMention) ccpMention;
883            ClassMention returnCM = new ClassMention();
884            returnCM.setMentionName(ccpClassMention.getMentionName());
885    
886            // for each slotMention, add a new Util Mention
887            FSArray ccpSlotMentionArray = ccpClassMention.getSlotMentions();
888            if (ccpSlotMentionArray != null) {
889                FeatureStructure[] ccpSlotMentions = ccpClassMention.getSlotMentions().toArray();
890                if (DEBUG) {
891                    System.out.print("...This mention is a ClassMention:  " + returnCM.getMentionName());
892                    System.out.println("... has " + ccpSlotMentions.length + " Knowtator slotMentions (complex and non-complex)");
893                }
894                for (FeatureStructure fs : ccpSlotMentions) {
895                    CCPSlotMention ccpSlotMention = (CCPSlotMention) fs;
896                    Mention mention = extractUtilMention(ccpSlotMention, jcas, alreadyExtractedAnnotations, alreadyExtractedMentions);
897                    if (mention instanceof ComplexSlotMention) {
898                        returnCM.addComplexSlotMention((ComplexSlotMention) mention);
899                    } else if (mention instanceof SlotMention) {
900                        returnCM.addSlotMention((SlotMention) mention);
901                    }
902                }
903            }
904    
905            // create other TextAnnotations associated with this knowtator mention
906            FSArray associatedSemanticAnnotationsArray = ccpClassMention.getCcpTextAnnotations();
907            if (associatedSemanticAnnotationsArray != null) {
908                FeatureStructure[] associatedSemanticAnnotations = ccpClassMention.getCcpTextAnnotations().toArray();
909                for (FeatureStructure fs : associatedSemanticAnnotations) {
910                    // SemanticAnnotation semanticAnnotation = (SemanticAnnotation)
911                    // fs;
912                    CCPTextAnnotation ccpTextAnnotation = (CCPTextAnnotation) fs;
913                    TextAnnotation ta = extractTextAnnotation(ccpTextAnnotation, jcas, alreadyExtractedAnnotations, alreadyExtractedMentions,
914                            returnCM);
915                    returnCM.addTextAnnotation(ta);
916                }
917            }
918            // we have now gotten a complete ClassMention back
919            // if it (or one like it) has already been stored, then use the one
920            // already stored, else store this new ClassMention.
921            String hashkey = returnCM.getSingleLineRepresentation();
922            if (alreadyExtractedMentions.containsKey(hashkey)) {
923                returnCM = alreadyExtractedMentions.get(hashkey);
924            } else {
925                alreadyExtractedMentions.put(hashkey, returnCM);
926            }
927    
928            return returnCM;
929        }
930    
931        private ComplexSlotMention processCCPComplexSlotMention(CCPMention ccpMention, JCas jcas,
932                HashMap<String, TextAnnotation> alreadyExtractedAnnotations, HashMap<String, ClassMention> alreadyExtractedMentions) {
933    
934            CCPComplexSlotMention ccpComplexSlotMention = (CCPComplexSlotMention) ccpMention;
935            ComplexSlotMention returnCSM = new ComplexSlotMention();
936            returnCSM.setMentionName(ccpComplexSlotMention.getMentionName());
937    
938            if (DEBUG) {
939                System.out.print("...This mention is a ComplexSlotMention:  " + returnCSM.getMentionName());
940            }
941    
942            FeatureStructure[] ccpClassMentions = ccpComplexSlotMention.getClassMentions().toArray();
943            for (FeatureStructure fs : ccpClassMentions) {
944                CCPClassMention ccpClassMention = (CCPClassMention) fs;
945                ClassMention cm = (ClassMention) extractUtilMention(ccpClassMention, jcas, alreadyExtractedAnnotations,
946                        alreadyExtractedMentions);
947                returnCSM.addClassMention(cm);
948            }
949            return returnCSM;
950        }
951    
952        private SlotMention processCCPSlotMention(CCPMention ccpMention, JCas jcas,
953                HashMap<String, TextAnnotation> alreadyExtractedAnnotations, HashMap<String, ClassMention> alreadyExtractedMentions) {
954    
955            CCPNonComplexSlotMention ccpNonComplexSlotMention = (CCPNonComplexSlotMention) ccpMention;
956            SlotMention returnSM = new SlotMention();
957            returnSM.setMentionName(ccpNonComplexSlotMention.getMentionName());
958    
959            if (DEBUG) {
960                System.out.println("...This mention is a SlotMention:  " + returnSM.getMentionName());
961            }
962    
963            String[] slotValues = ccpNonComplexSlotMention.getSlotValues().toArray();
964            for (String value : slotValues) {
965                ((SlotMention) returnSM).addSlotValue(value);
966            }
967            return returnSM;
968        }
969    
970        private String getCCPTextAnnotationHashKey(CCPTextAnnotation ccpTextAnnotation) {
971            return getSingleLineRepresentationOfCCPTextAnnotation(ccpTextAnnotation, true);
972            // String key = "";
973            //
974            // // get the spans of text associated with this annotation
975            // FeatureStructure[] ccpSpans = ccpTextAnnotation.getSpans().toArray();
976            // for (FeatureStructure fs : ccpSpans) {
977            // CCPSpan ccpSpan = (CCPSpan) fs;
978            // key += (Integer.toString(ccpSpan.getSpanStart()) + " -- " + Integer.toString(ccpSpan.getSpanEnd()) + " ");
979            // }
980            //
981            // int annotatorID = ccpTextAnnotation.getAnnotator().getAnnotatorID();
982            // key += (" " + annotatorID);
983            //
984            // // get the mention for this annotation
985            // CCPClassMention ccpClassMention = ccpTextAnnotation.getClassMention();
986            //
987            // try {
988            // key += (" " + ccpClassMention.getMentionName());
989            // } catch (Exception e) {
990            // System.err.println("Found ccpTextAnnotation without a class mention");
991            // printCCPTextAnnotation(ccpTextAnnotation, System.out);
992            // e.printStackTrace();
993            // }
994            //
995            // return key;
996        }
997    
998        public void putTextAnnotationsIntoJCas(JCas jcas, List<TextAnnotation> textAnnotations) {
999    
1000            // System.err.println("DEBUG -- UIMA_Util: About to add " + textAnnotations.size() + " into CAS...");
1001            // System.err.println("DEBUG -- UIMA_Util: Currently: " +
1002            // jcas.getJFSIndexRepository().getAnnotationIndex(CCPTextAnnotation.type).size() + " annotations in CAS.");
1003    
1004            // Iterator annotIter = jcas.getJFSIndexRepository().getAnnotationIndex(CCPTextAnnotation.type).iterator();
1005            // while (annotIter.hasNext()) {
1006            // CCPTextAnnotation ccpTA = (CCPTextAnnotation) annotIter.next();
1007            // System.out.println("DEBUG -- UIMA_Util: Annotations in CAS...");
1008            // UIMA_Util.printCCPTextAnnotation(ccpTA, System.out);
1009            // }
1010    
1011            HashMap<String, String> alreadyCreatedAnnotations = new HashMap<String, String>();
1012            HashMap<String, CCPClassMention> alreadyCreatedMentions = new HashMap<String, CCPClassMention>();
1013    
1014            for (TextAnnotation ta : textAnnotations) {
1015    
1016                // System.out.println("DEBUG -- UIMA_Util: Putting annotation into CAS...");
1017                // System.out.println("SINGLE LINE REP: " + ta.getSingleLineRepresentation(false, true));
1018                // ta.printAnnotation(System.out);
1019    
1020                createUIMAAnnotation(ta, jcas, alreadyCreatedAnnotations, alreadyCreatedMentions, null);
1021            }
1022    
1023            if (DEBUG) {
1024                System.out.println("#############################################");
1025                System.out.println("# of alreadyCreatedAnnotations = " + alreadyCreatedAnnotations.size());
1026                Iterator keyIter = alreadyCreatedAnnotations.keySet().iterator();
1027                while (keyIter.hasNext()) {
1028                    System.out.println("annotation key: " + keyIter.next());
1029                }
1030                System.out.println("# of alreadyCreatedMentions = " + alreadyCreatedMentions.size());
1031                keyIter = alreadyCreatedMentions.keySet().iterator();
1032                while (keyIter.hasNext()) {
1033                    System.out.println("annotation key: " + keyIter.next());
1034                }
1035                System.out.println("#############################################");
1036            }
1037    
1038            // System.err.println("DEBUG -- UIMA_Util: Added " + jcas.getJFSIndexRepository().getAnnotationIndex(CCPTextAnnotation.type).size()
1039            // + " into CAS.");
1040            // Iterator annotIter = jcas.getJFSIndexRepository().getAnnotationIndex(CCPTextAnnotation.type).iterator();
1041            // while (annotIter.hasNext()) {
1042            // CCPTextAnnotation ccpTA = (CCPTextAnnotation) annotIter.next();
1043            // System.out.println("DEBUG -- UIMA_Util: Annotations in CAS...");
1044            // UIMA_Util.printCCPTextAnnotation(ccpTA, System.out);
1045            // }
1046    
1047        }
1048    
1049        /** ********************************************************************************************************************************* */
1050    
1051        // /**
1052        // *
1053        // * @param ta
1054        // * @param alreadyCreatedAnnotations
1055        // * @param alreadyCreatedMentions
1056        // * @param mention
1057        // * if this is null, a new mention is created from
1058        // * ta.getClassMention, otherwise, this inputed mention is used
1059        // * for creating the Knowtator annotation
1060        // */
1061        // private void createUIMAAnnotation(TextAnnotation ta, JCas jcas,
1062        // HashMap<String, String> alreadyCreatedAnnotations,
1063        // HashMap<String, CCPClassMention> alreadyCreatedMentions, CCPClassMention
1064        // classMention,
1065        // AnnotationSetValidator annotationSetValidator, AnnotatorValidator
1066        // annotatorValidator) {
1067        //
1068        // // check to see if the text annotation has already been added
1069        // if (!alreadyCreatedAnnotations.containsKey(ta.getHashKey())) {
1070        //
1071        // /*
1072        // * Is this a syntactic annotation, i.e. is it a Token, Phrase, or
1073        // * Clause? If it is, then create the corresponding UIMA annotation.
1074        // * If it is not, then create a UIMA semantic annotation.
1075        // */
1076        // edu.uchsc.ccp.util.nlp.mention.ClassMention cm = ta.getClassMention();
1077        // if (!(cm == null) && cm.getMentionName().equalsIgnoreCase("token")) {
1078        // addUIMATokenAnnotation(ta, jcas, alreadyCreatedAnnotations);
1079        // } else if (!(cm == null) &&
1080        // cm.getMentionName().equalsIgnoreCase("phrase")) {
1081        // addUIMAPhraseAnnotation(ta, jcas, alreadyCreatedAnnotations);
1082        // } else if (!(cm == null) &&
1083        // cm.getMentionName().equalsIgnoreCase("clause")) {
1084        // addUIMAClauseAnnotation(ta, jcas, alreadyCreatedAnnotations);
1085        // } else { // we are dealing with some kind of semantic annotation
1086        // addUIMASemanticAnnotation(ta, classMention, jcas,
1087        // alreadyCreatedAnnotations, alreadyCreatedMentions);
1088        // }
1089        // } else {
1090        // // do nothing, this annotation has already been created in the
1091        // // Knowtator project
1092        // }
1093        //
1094        // }
1095        // private void addUIMATokenAnnotation(TextAnnotation ta, JCas jcas,
1096        // HashMap<String, String> alreadyCreatedAnnotations) {
1097        // // create a new TokenAnnotation
1098        // TokenAnnotation tokenAnnotation = new TokenAnnotation(jcas);
1099        //
1100        // // extract the annotation information from the TextAnnotation and
1101        // // fill the corresponding fields in the TokenAnnotation
1102        // UIMA_Util.swapAnnotationInfo(ta, tokenAnnotation, jcas);
1103        // ClassMention cm = ta.getClassMention();
1104        // // get POS
1105        // SlotMention posSlot = cm.getSlotMentionByName("partOfSpeech");
1106        // if (posSlot != null) {
1107        // String posLabel = (String) posSlot.getSlotValues().get(0);
1108        // tokenAnnotation.setPartOfSpeech(posLabel);
1109        // } else {
1110        // tokenAnnotation.setPartOfSpeech(null);
1111        // }
1112        //
1113        // // get Lemma
1114        // SlotMention lemmaSlot = cm.getSlotMentionByName("lemma");
1115        // if (lemmaSlot != null) {
1116        // String lemmaLabel = (String) lemmaSlot.getSlotValues().get(0);
1117        // tokenAnnotation.setLemma(lemmaLabel);
1118        // } else {
1119        // tokenAnnotation.setLemma(null);
1120        // }
1121        //
1122        // // get Stem
1123        // SlotMention stemSlot = cm.getSlotMentionByName("stem");
1124        // if (stemSlot != null) {
1125        // String stemLabel = (String) stemSlot.getSlotValues().get(0);
1126        // tokenAnnotation.setStem(stemLabel);
1127        // } else {
1128        // tokenAnnotation.setStem(null);
1129        // }
1130        //
1131        // // add key to alreadyAddedAnnotations
1132        // alreadyCreatedAnnotations.put(ta.getHashKey(), "");
1133        //
1134        // // add TokenAnnotation to CAS indexes
1135        // tokenAnnotation.addToIndexes();
1136        // }
1137        //
1138        // private void addUIMAPhraseAnnotation(TextAnnotation ta, JCas jcas,
1139        // HashMap<String, String> alreadyCreatedAnnotations) {
1140        // PhraseAnnotation phraseAnnotation = new PhraseAnnotation(jcas);
1141        //
1142        // // extract the annotation information from the TextAnnotation and
1143        // // fill the corresponding fields in the PhraseAnnotation
1144        // UIMA_Util.swapAnnotationInfo(ta, phraseAnnotation, jcas);
1145        //
1146        // ClassMention cm = ta.getClassMention();
1147        // // get POS
1148        // SlotMention phraseTypeSlot = cm.getSlotMentionByName("phraseType");
1149        // if (phraseTypeSlot != null) {
1150        // String phraseTypeLabel = (String) phraseTypeSlot.getSlotValues().get(0);
1151        // phraseAnnotation.setPhraseType(phraseTypeLabel);
1152        // } else {
1153        // phraseAnnotation.setPhraseType(null);
1154        // }
1155        //
1156        // // add key to alreadyAddedAnnotations
1157        // alreadyCreatedAnnotations.put(ta.getHashKey(), "");
1158        //
1159        // // add PhraseAnnotation to CAS indexes
1160        // phraseAnnotation.addToIndexes();
1161        //
1162        // }
1163        //
1164        // private void addUIMAClauseAnnotation(TextAnnotation ta, JCas jcas,
1165        // HashMap<String, String> alreadyCreatedAnnotations) {
1166        // ClauseAnnotation clauseAnnotation = new ClauseAnnotation(jcas);
1167        //
1168        // // extract the annotation information from the TextAnnotation and
1169        // // fill the corresponding fields in the PhraseAnnotation
1170        // UIMA_Util.swapAnnotationInfo(ta, clauseAnnotation, jcas);
1171        //
1172        // ClassMention cm = ta.getClassMention();
1173        // // get POS
1174        // SlotMention clauseTypeSlot = cm.getSlotMentionByName("clauseType");
1175        // if (clauseTypeSlot != null) {
1176        // String clauseTypeLabel = (String) clauseTypeSlot.getSlotValues().get(0);
1177        // clauseAnnotation.setClauseType(clauseTypeLabel);
1178        // } else {
1179        // clauseAnnotation.setClauseType(null);
1180        // }
1181        //
1182        // // add key to alreadyAddedAnnotations
1183        // alreadyCreatedAnnotations.put(ta.getHashKey(), "");
1184        //
1185        // // add PhraseAnnotation to CAS indexes
1186        // clauseAnnotation.addToIndexes();
1187        // }
1188        // private void addUIMASemanticAnnotation(TextAnnotation ta, CCPClassMention
1189        // classMention, JCas jcas,
1190        // HashMap<String, String> alreadyCreatedAnnotations, HashMap<String,
1191        // CCPClassMention> alreadyCreatedMentions) {
1192        private void createUIMAAnnotation(TextAnnotation ta, JCas jcas, HashMap<String, String> alreadyCreatedAnnotations,
1193                HashMap<String, CCPClassMention> alreadyCreatedMentions, CCPClassMention classMention) {
1194    
1195            if (!alreadyCreatedAnnotations.containsKey(ta.getSingleLineRepresentation(false, true))) {
1196    
1197                // /*
1198                // * Is this a syntactic annotation, i.e. is it a Token, Phrase, or
1199                // * Clause? If it is, then create the corresponding UIMA
1200                // annotation.
1201                // * If it is not, then create a UIMA semantic annotation.
1202                // */
1203                // edu.uchsc.ccp.util.nlp.mention.ClassMention cm =
1204                // ta.getClassMention();
1205                // if (!(cm == null) &&
1206                // cm.getMentionName().equalsIgnoreCase("token")) {
1207                // addUIMATokenAnnotation(ta, jcas, alreadyCreatedAnnotations);
1208                // } else if (!(cm == null) &&
1209                // cm.getMentionName().equalsIgnoreCase("phrase")) {
1210                // addUIMAPhraseAnnotation(ta, jcas, alreadyCreatedAnnotations);
1211                // } else if (!(cm == null) &&
1212                // cm.getMentionName().equalsIgnoreCase("clause")) {
1213                // addUIMAClauseAnnotation(ta, jcas, alreadyCreatedAnnotations);
1214                // } else { // we are dealing with some kind of semantic annotation
1215                // addUIMASemanticAnnotation(ta, classMention, jcas,
1216                // alreadyCreatedAnnotations, alreadyCreatedMentions);
1217                // }
1218    
1219                CCPTextAnnotation ccpTextAnnotation = new CCPTextAnnotation(jcas);
1220    
1221                // extract the annotation information from the TextAnnotation and
1222                // fill the corresponding fields in the SemanticAnnotation
1223                UIMA_Util.swapAnnotationInfo(ta, ccpTextAnnotation, jcas);
1224    
1225                // add key to alreadyAddedAnnotations
1226                alreadyCreatedAnnotations.put(ta.getSingleLineRepresentation(false, true), "");
1227    
1228                // if mention is null, then create a new UIMA mention from the
1229                // associated ClassMention, else, use mention
1230                // this prevents infinite loops from occurring when creating an
1231                // annotation from a ClassMention that was found in a
1232                // complexSlotMention.
1233                if (classMention == null) {
1234                    // create associated class mention
1235                    ClassMention cm = ta.getClassMention();
1236                    classMention = (CCPClassMention) this.createUIMAMention(cm, jcas, alreadyCreatedAnnotations, alreadyCreatedMentions);
1237                }
1238    
1239                
1240    //            /* Made a change here for UIMA 2.0..  getting TextAnnotations used to return null, but now throws an exception if there are none to return. */
1241    //            FSArray fsa;
1242    //            try {
1243    //            FSArray associatedSemanticAnnotations = classMention.getCcpTextAnnotations();
1244    //            fsa = new FSArray(jcas, associatedSemanticAnnotations.size() + 1);
1245    //            for (int i = 0; i < associatedSemanticAnnotations.size(); i++) {
1246    //                fsa.set(i, associatedSemanticAnnotations.get(i));
1247    //            }
1248    //            fsa.set(fsa.size() - 1, ccpTextAnnotation);
1249    //            } catch (CASRuntimeException casre) {
1250    //               fsa = new FSArray(jcas, 1);
1251    //                 fsa.set(0, ccpTextAnnotation);
1252    //            }
1253                FSArray fsa;
1254                FSArray associatedSemanticAnnotations = classMention.getCcpTextAnnotations();
1255                if (associatedSemanticAnnotations == null) {
1256                    fsa = new FSArray(jcas, 1);
1257                    fsa.set(0, ccpTextAnnotation);
1258                } else {
1259                    fsa = new FSArray(jcas, associatedSemanticAnnotations.size() + 1);
1260                    for (int i = 0; i < associatedSemanticAnnotations.size(); i++) {
1261                        fsa.set(i, associatedSemanticAnnotations.get(i));
1262                    }
1263                    fsa.set(fsa.size() - 1, ccpTextAnnotation);
1264                }
1265                classMention.setCcpTextAnnotations(fsa);
1266    
1267                
1268                ccpTextAnnotation.setClassMention(classMention);
1269    
1270                // add the SemanticAnnotation to the CAS indexes
1271                ccpTextAnnotation.addToIndexes();
1272            } else {
1273                // do nothing, this annotation has already been created in the
1274                // Knowtator project
1275            }
1276        }
1277    
1278        private CCPMention createUIMAMention(Mention mentionToAdd, JCas jcas, HashMap<String, String> alreadyCreatedAnnotations,
1279                HashMap<String, CCPClassMention> alreadyCreatedMentions) {
1280    
1281            CCPMention returnMention;
1282    
1283            if (DEBUG) {
1284                System.out.println("Creating new Knowtator mention: " + mentionToAdd.getMentionName());
1285            }
1286    
1287            if (mentionToAdd instanceof ClassMention) {
1288                returnMention = processClassMention((ClassMention) mentionToAdd, jcas, alreadyCreatedAnnotations, alreadyCreatedMentions);
1289            } else if (mentionToAdd instanceof ComplexSlotMention) {
1290                returnMention = processComplexSlotMention((ComplexSlotMention) mentionToAdd, jcas, alreadyCreatedAnnotations,
1291                        alreadyCreatedMentions);
1292            } else if (mentionToAdd instanceof SlotMention) {
1293                returnMention = processSlotMention((SlotMention) mentionToAdd, jcas, alreadyCreatedAnnotations, alreadyCreatedMentions);
1294            } else {
1295                System.err.println("The mention you are trying to create is an instance of: " + mentionToAdd.getClass().getName());
1296                System.err.println("Currently, only ClassMentions, ComplexSlotMentions, and SlotMentions can be added.");
1297                returnMention = null;
1298            }
1299    
1300            return returnMention;
1301        }
1302    
1303        private CCPClassMention processClassMention(ClassMention classMention, JCas jcas, HashMap<String, String> alreadyCreatedAnnotations,
1304                HashMap<String, CCPClassMention> alreadyCreatedMentions) {
1305    
1306            CCPClassMention ccpClassMention;
1307    
1308            // check to see if this mention has already been created
1309            if (alreadyCreatedMentions.containsKey(classMention.getSingleLineRepresentation())) {
1310                ccpClassMention = alreadyCreatedMentions.get(classMention.getSingleLineRepresentation());
1311            } else {
1312                // create a new Knowtator mention
1313    
1314                ccpClassMention = new CCPClassMention(jcas);
1315                ccpClassMention.setMentionName(classMention.getMentionName());
1316    
1317                /**
1318                 * should this be at the end??? No, it doesn't matter because classMention.getHashKey is complete
1319                 */
1320                // add the knowtator to the alreadyCreatedMentions hash
1321                alreadyCreatedMentions.put(classMention.getSingleLineRepresentation(), ccpClassMention);
1322    
1323                // for each complexSlotMention, add a new knowtator class
1324                // mention
1325                List<ComplexSlotMention> complexSlotMentions = classMention.getComplexSlotMentions();
1326                List<SlotMention> slotMentions = classMention.getSlotMentions();
1327    
1328                if (DEBUG) {
1329                    System.out.println("...This mention is a ClassMention");
1330                    System.out.println("... has " + complexSlotMentions.size() + " ComplexSlotMentions");
1331                }
1332    
1333                FSArray slotMentionFSArray = new FSArray(jcas, complexSlotMentions.size() + slotMentions.size());
1334                int fsArrayIndex = 0;
1335                for (ComplexSlotMention csm : complexSlotMentions) {
1336                    CCPComplexSlotMention ccpComplexSlotMention = (CCPComplexSlotMention) createUIMAMention(csm, jcas,
1337                            alreadyCreatedAnnotations, alreadyCreatedMentions);
1338    
1339                    if (DEBUG) {
1340                        System.out.println("......adding slot: " + csm.getMentionName() + "  to: " + classMention.getMentionName());
1341                    }
1342    
1343                    slotMentionFSArray.set(fsArrayIndex++, ccpComplexSlotMention);
1344                }
1345    
1346                // for each (non-complex) slot mention
1347    
1348                if (DEBUG) {
1349                    System.out.println("... has " + slotMentions.size() + " SlotMentions");
1350                }
1351    
1352                for (SlotMention sm : slotMentions) {
1353                    CCPNonComplexSlotMention ccpNonComplexSlotMention = (CCPNonComplexSlotMention) createUIMAMention(sm, jcas,
1354                            alreadyCreatedAnnotations, alreadyCreatedMentions);
1355    
1356                    if (DEBUG) {
1357                        System.out.println("......adding slot: " + sm.getMentionName() + "  to: " + classMention.getMentionName());
1358                    }
1359    
1360                    slotMentionFSArray.set(fsArrayIndex++, ccpNonComplexSlotMention);
1361                }
1362    
1363                // add slot mentions array to classmention
1364                ccpClassMention.setSlotMentions(slotMentionFSArray);
1365    
1366                // create Knowtator annotation of TextAnnotation(s) associated with
1367                // this ClassMention
1368                List<TextAnnotation> associatedTextAnnotations = classMention.getTextAnnotations();
1369                for (TextAnnotation ta : associatedTextAnnotations) {
1370                    createUIMAAnnotation(ta, jcas, alreadyCreatedAnnotations, alreadyCreatedMentions, ccpClassMention);
1371                }
1372            }
1373            return ccpClassMention;
1374        }
1375    
1376        private CCPComplexSlotMention processComplexSlotMention(ComplexSlotMention complexSlotMention, JCas jcas,
1377                HashMap<String, String> alreadyCreatedAnnotations, HashMap<String, CCPClassMention> alreadyCreatedMentions) {
1378    
1379            if (DEBUG) {
1380                System.out.println("...This mention is a ComplexSlotMention");
1381            }
1382    
1383            // create a new Knowtator mention
1384            CCPComplexSlotMention ccpComplexSlotMention = new CCPComplexSlotMention(jcas);
1385            ccpComplexSlotMention.setMentionName(complexSlotMention.getMentionName());
1386    
1387            // for each of its classMentions, create a new Knowtator mention
1388            List<ClassMention> classMentions = complexSlotMention.getClassMentions();
1389    
1390            FSArray classMentionFSArray = new FSArray(jcas, classMentions.size());
1391            int fsArrayIndex = 0;
1392    
1393            for (ClassMention cm : classMentions) {
1394                CCPClassMention ccpClassMention = (CCPClassMention) createUIMAMention(cm, jcas, alreadyCreatedAnnotations,
1395                        alreadyCreatedMentions);
1396    
1397                if (DEBUG) {
1398                    System.out.println("......adding value: " + cm.getMentionName() + "  to slot mention: "
1399                            + complexSlotMention.getMentionName());
1400                }
1401    
1402                classMentionFSArray.set(fsArrayIndex++, ccpClassMention);
1403            }
1404    
1405            // add slot mentions array to classmention
1406            ccpComplexSlotMention.setClassMentions(classMentionFSArray);
1407    
1408            return ccpComplexSlotMention;
1409        }
1410    
1411        private CCPNonComplexSlotMention processSlotMention(SlotMention slotMention, JCas jcas,
1412                HashMap<String, String> alreadyCreatedAnnotations, HashMap<String, CCPClassMention> alreadyCreatedMentions) {
1413    
1414            if (DEBUG) {
1415                System.out.println("...This mention is a SlotMention");
1416            }
1417    
1418            // create a new Knowtator mention
1419            CCPNonComplexSlotMention ccpNonComplexSlotMention = new CCPNonComplexSlotMention(jcas);
1420            ccpNonComplexSlotMention.setMentionName(slotMention.getMentionName());
1421    
1422            // for each Object slot value, add it to the Knowtator slot mention
1423            ArrayList<Object> slotValues = (ArrayList<Object>) slotMention.getSlotValues();
1424    
1425            StringArray valueArray = new StringArray(jcas, slotValues.size());
1426            int stringArrayIndex = 0;
1427    
1428            // we are making an assumption that the slotValues are Strings
1429            for (Object value : slotValues) {
1430                valueArray.set(stringArrayIndex++, (String) value.toString());
1431            }
1432    
1433            ccpNonComplexSlotMention.setSlotValues(valueArray);
1434    
1435            return ccpNonComplexSlotMention;
1436        }
1437    
1438        public static void printCCPAnnotator(CCPAnnotator ccpAnnotator, PrintStream ps) {
1439            String annotatorStr = ccpAnnotator.getAnnotatorID() + " - " + ccpAnnotator.getFirstName() + " " + ccpAnnotator.getLastName()
1440                    + " -- " + ccpAnnotator.getAffiliation();
1441            ps.println(annotatorStr);
1442        }
1443    
1444        public static void printCCPAnnotationSet(CCPAnnotationSet ccpAnnotationSet, PrintStream ps) {
1445            String annotationSetStr = ccpAnnotationSet.getAnnotationSetID() + " - " + ccpAnnotationSet.getAnnotationSetName() + " -- "
1446                    + ccpAnnotationSet.getAnnotationSetDescription();
1447            ps.println(annotationSetStr);
1448        }
1449    
1450        public String getSingleLineRepresentationOfCCPTextAnnotation(CCPTextAnnotation ccpTA, boolean deep) {
1451            String repStr = "";
1452            ;
1453    
1454            // if (showDocID) {
1455            // repStr = documentID + "|" ;
1456            // } else {
1457            // repStr = "";
1458            // }
1459    
1460            String spansStr = "";
1461            FSArray spans = ccpTA.getSpans();
1462            if (spans != null) {
1463                for (int i = 0; i < spans.size(); i++) {
1464                    CCPSpan span = (CCPSpan) spans.get(i);
1465                    spansStr += ("[" + span.getSpanStart() + ".." + span.getSpanEnd() + "]");
1466                }
1467                spansStr = spansStr.substring(0, spansStr.length());
1468            }
1469    
1470            String coveredText;
1471            try {
1472                coveredText = ccpTA.getCoveredText();
1473            } catch (StringIndexOutOfBoundsException siobe) {
1474                coveredText = "";
1475            }
1476            CCPAnnotator ccpAnnotator = ccpTA.getAnnotator();
1477            String lastName = "";
1478            if (ccpAnnotator != null) {
1479                lastName = ccpAnnotator.getLastName();
1480            }
1481    
1482            String annotationSetStr = "";
1483            FSArray ccpAnnotationSets = ccpTA.getAnnotationSets();
1484            if (ccpAnnotationSets != null) {
1485                for (int i = 0; i < ccpAnnotationSets.size(); i++) {
1486                    CCPAnnotationSet ccpASet = (CCPAnnotationSet) ccpAnnotationSets.get(i);
1487                    annotationSetStr += (ccpASet.getAnnotationSetID() + ",");
1488                }
1489            }
1490    
1491            if (deep) {
1492                repStr += (spansStr + "|\"" + coveredText + "\"" + "|" + lastName + "|" + annotationSetStr + "{"
1493                        + getSingleLineRepresentationOfCCPClassMention(ccpTA.getClassMention()) + "}");
1494            } else {
1495                repStr += (spansStr + "|\"" + coveredText + "\"" + "|" + lastName + "|" + annotationSetStr);
1496            }
1497            return repStr;
1498        }
1499    
1500        public String getSingleLineRepresentationOfCCPNonComplexSlotMention(CCPNonComplexSlotMention ccpNCSM) {
1501            String repStr = " [" + ccpNCSM.getMentionName() + "]:";
1502    
1503            StringArray slotValues = ccpNCSM.getSlotValues();
1504            for (int i = 0; i < slotValues.size(); i++) {
1505                repStr += slotValues.get(i) + ":";
1506            }
1507            repStr = repStr.substring(0, repStr.length() - 1);
1508            return repStr;
1509    
1510        }
1511    
1512        public String getSingleLineRepresentationOfCCPComplexSlotMention(CCPComplexSlotMention ccpCSM) {
1513            String repStr = " [" + ccpCSM.getMentionName() + "]:";
1514            FSArray classMentions = ccpCSM.getClassMentions();
1515            for (int i = 0; i < classMentions.size(); i++) {
1516                CCPClassMention ccpCM = (CCPClassMention) classMentions.get(i);
1517                FSArray associatedTextAnnotations = ccpCM.getCcpTextAnnotations();
1518                repStr += getSingleLineRepresentationOfCCPTextAnnotation((CCPTextAnnotation) associatedTextAnnotations.get(0), true);
1519            }
1520            return repStr;
1521    
1522        }
1523    
1524        public String getSingleLineRepresentationOfCCPClassMention(CCPClassMention ccpCM) {
1525            if (ccpCM != null) {
1526                FSArray associatedTextAnnotations = ccpCM.getCcpTextAnnotations();
1527    
1528                String repStr = " [" + ccpCM.getMentionName() + "]";
1529                if (associatedTextAnnotations == null) {
1530                    System.err.println("ERROR -- UIMA_Util: FOUND ClassMention without a reference to its TextAnnotation...");
1531                    UIMA_Util.printCCPClassMention(ccpCM, System.err, 0);
1532                    return null;
1533                } else {
1534                    repStr += getSingleLineRepresentationOfCCPTextAnnotation((CCPTextAnnotation) associatedTextAnnotations.get(0), false) + "(";
1535                }
1536    
1537                FSArray ccpSlotMentions = ccpCM.getSlotMentions();
1538                List<CCPSlotMention> ccpSlotMentionsList = new ArrayList<CCPSlotMention>();
1539                if (ccpSlotMentions != null) {
1540                    for (int i = 0; i < ccpSlotMentions.size(); i++) {
1541                        ccpSlotMentionsList.add((CCPSlotMention) ccpSlotMentions.get(i));
1542                    }
1543                }
1544    
1545                List<CCPSlotMention> sortedSlotMentions = sortSlotMentions(ccpSlotMentionsList);
1546                for (CCPSlotMention sm : sortedSlotMentions) {
1547                    repStr += getSingleLineRepresentationOfCCPSlotMention(sm);
1548                }
1549    
1550                repStr += ")";
1551                return repStr;
1552            } else {
1553                return null;
1554            }
1555        }
1556    
1557        private String getSingleLineRepresentationOfCCPSlotMention(CCPSlotMention ccpSM) {
1558            if (ccpSM instanceof CCPNonComplexSlotMention) {
1559                return getSingleLineRepresentationOfCCPNonComplexSlotMention((CCPNonComplexSlotMention) ccpSM);
1560            } else if (ccpSM instanceof CCPComplexSlotMention) {
1561                return getSingleLineRepresentationOfCCPComplexSlotMention((CCPComplexSlotMention) ccpSM);
1562            } else {
1563                System.err.println("Expecting CCPNonComplexSlotMention or CCPComplexSlotMention but instead got " + ccpSM.getClass().getName());
1564                return null;
1565            }
1566        }
1567    
1568        private List<CCPSlotMention> sortSlotMentions(List<CCPSlotMention> slotMentions) {
1569            List<CCPSlotMention> sortedCSMList = new ArrayList<CCPSlotMention>();
1570            List<CCPSlotMention> sortedSMList = new ArrayList<CCPSlotMention>();
1571    
1572            HashMap<String, CCPSlotMention> inputCSMHash = new HashMap<String, CCPSlotMention>();
1573            HashMap<String, CCPSlotMention> inputSMHash = new HashMap<String, CCPSlotMention>();
1574    
1575            for (CCPSlotMention ment : slotMentions) {
1576                if (ment instanceof CCPComplexSlotMention) {
1577                    inputCSMHash.put(getSingleLineRepresentationOfCCPSlotMention(ment), ment);
1578                } else if (ment instanceof CCPNonComplexSlotMention) {
1579                    inputSMHash.put(getSingleLineRepresentationOfCCPSlotMention(ment), ment);
1580                } else {
1581                    System.err.println("Expecting sm or csm in uima_util.sortSLotMEntions!!!!!");
1582                }
1583    
1584            }
1585    
1586            Set<String> keys = inputCSMHash.keySet();
1587            List<String> sortedKeys = Arrays.asList(keys.toArray(new String[0]));
1588            for (String key : sortedKeys) {
1589                sortedCSMList.add(inputCSMHash.get(key));
1590            }
1591    
1592            keys = inputSMHash.keySet();
1593            sortedKeys = Arrays.asList(keys.toArray(new String[0]));
1594            for (String key : sortedKeys) {
1595                sortedSMList.add(inputSMHash.get(key));
1596            }
1597    
1598            sortedCSMList.addAll(sortedSMList);
1599            return sortedCSMList;
1600    
1601        }
1602    
1603        public static void printCCPTextAnnotation(CCPTextAnnotation ccpTextAnnotation, PrintStream ps) {
1604            String spansStr = "";
1605            FSArray spans = ccpTextAnnotation.getSpans();
1606            for (int i = 0; i < spans.size(); i++) {
1607                CCPSpan span = (CCPSpan) spans.get(i);
1608                spansStr += (span.getSpanStart() + " - " + span.getSpanEnd() + "  ");
1609            }
1610    
1611            ps.println("======================= Annotation: " + ccpTextAnnotation.getAnnotationID() + " =======================");
1612            ps.print("Annotator: ");
1613            printCCPAnnotator(ccpTextAnnotation.getAnnotator(), ps);
1614            ps.print("--- AnnotationSets: ");
1615            FSArray annotationSets = ccpTextAnnotation.getAnnotationSets();
1616            if (annotationSets != null) {
1617                for (int i = 0; i < annotationSets.size(); i++) {
1618                    printCCPAnnotationSet((CCPAnnotationSet) annotationSets.get(i), ps);
1619                }
1620                if (annotationSets.size() == 0) {
1621                    ps.println();
1622                }
1623            } else {
1624                ps.println();
1625            }
1626            ps.println("--- Default Span: " + ccpTextAnnotation.getBegin() + " - " + ccpTextAnnotation.getEnd());
1627            ps.println("--- Span: " + spansStr);
1628            ps.println("--- DocumentSection: " + ccpTextAnnotation.getDocumentSectionID());
1629            ps.println("--- Covered Text: " + ccpTextAnnotation.getCoveredText());
1630    
1631            CCPClassMention ccpCM = ccpTextAnnotation.getClassMention();
1632            if (ccpCM != null) {
1633                printCCPClassMention(ccpCM, ps, 0);
1634            } else {
1635                ps.println("--- ClassMention: NULL");
1636            }
1637            ps.println("=================================================================================");
1638    
1639        }
1640    
1641        public static void printCCPClassMention(CCPClassMention ccpClassMention, PrintStream ps, int indentLevel) {
1642            String indentSpace = "-";
1643            for (int i = 0; i < indentLevel * 4; i++) {
1644                indentSpace += " ";
1645            }
1646            if (ccpClassMention != null) {
1647                // Print out class mention name and span string
1648                ps.println(indentSpace + "CLASS MENTION: " + ccpClassMention.getMentionName() + " \""
1649                        + getCCPClassMentionString(ccpClassMention) + "\"");
1650                indentLevel++;
1651    
1652                try {
1653                    FeatureStructure[] slotMentions = ccpClassMention.getSlotMentions().toArray();
1654                    for (FeatureStructure slotMention : slotMentions) {
1655                        if (slotMention instanceof CCPComplexSlotMention) {
1656                            printCCPComplexSlotMention((CCPComplexSlotMention) slotMention, ps, indentLevel);
1657                        } else if (slotMention instanceof CCPNonComplexSlotMention) {
1658                            printCCPNonComplexSlotMention((CCPNonComplexSlotMention) slotMention, ps, indentLevel);
1659                        } else {
1660                            System.err
1661                                    .println("Error while trying to print slot mention of CCPClassMention - it was neither a CCPComplexSlotMention nor a CCPNonComplexSlotMention. Instead it was a "
1662                                            + slotMention.getClass().getName());
1663                        }
1664                    }
1665                } catch (NullPointerException npe) {
1666                    // if the CCPClassMention has not SlotMentions, then we end up
1667                    // here
1668                    // so, do nothing.
1669                }
1670            } else {
1671                ps.println(indentSpace + "CLASS MENTION: null");
1672            }
1673        }
1674    
1675        public static void printCCPComplexSlotMention(CCPComplexSlotMention ccpComplexSlotMention, PrintStream ps, int indentLevel) {
1676            String indentSpace = "-";
1677            for (int i = 0; i < indentLevel * 4; i++) {
1678                indentSpace += " ";
1679            }
1680            ps.println(indentSpace + "COMPLEX SLOT MENTION: " + ccpComplexSlotMention.getMentionName());
1681            indentLevel++;
1682    
1683            FeatureStructure[] classMentions = ccpComplexSlotMention.getClassMentions().toArray();
1684            for (FeatureStructure classMention : classMentions) {
1685                if (classMention instanceof CCPClassMention) {
1686                    printCCPClassMention((CCPClassMention) classMention, ps, indentLevel);
1687                } else {
1688                    System.err
1689                            .println("Error while trying to print slot mention of CCPComplexSlotMention - it was not a CCPClassMention. Instead it was a "
1690                                    + classMention.getClass().getName());
1691                }
1692            }
1693        }
1694    
1695        public static void printCCPNonComplexSlotMention(CCPNonComplexSlotMention ccpNonComplexSlotMention, PrintStream ps, int indentLevel) {
1696            String indentSpace = "-";
1697            for (int i = 0; i < indentLevel * 4; i++) {
1698                indentSpace += " ";
1699            }
1700            ps.print(indentSpace + "SLOT MENTION: " + ccpNonComplexSlotMention.getMentionName() + " with SLOT VALUE(s): ");
1701            String[] values = ccpNonComplexSlotMention.getSlotValues().toArray();
1702            for (String value : values) {
1703                ps.print(value + "  ");
1704            }
1705            ps.println();
1706        }
1707    
1708        public static CCPSlotMention getSlotMentionByName(CCPTextAnnotation ccpTextAnnotation, String slotMentionName) {
1709            return getSlotMentionByName(ccpTextAnnotation.getClassMention(), slotMentionName);
1710        }
1711    
1712        public static CCPSlotMention getSlotMentionByName(CCPClassMention ccpClassMention, String slotMentionName) {
1713            FSArray slotMentionsArray = ccpClassMention.getSlotMentions();
1714            if (slotMentionsArray != null) {
1715                CCPSlotMention returnSlotMention = null;
1716                for (int i = 0; i < slotMentionsArray.size(); i++) {
1717                    // FeatureStructure[] slotMentions = slotMentionsArray.toArray();
1718                    // for (FeatureStructure fs : slotMentions) {
1719                    FeatureStructure fs = slotMentionsArray.get(i);
1720                    if (fs instanceof CCPSlotMention) {
1721                        CCPSlotMention ccpSlotMention = (CCPSlotMention) fs;
1722                        if (ccpSlotMention.getMentionName().equals(slotMentionName)) {
1723                            returnSlotMention = ccpSlotMention;
1724                            break;
1725                        }
1726                    } else {
1727                        error("Expecting CCPSlotMention but got a : " + fs.getClass().getName());
1728                    }
1729                }
1730                return returnSlotMention;
1731            } else {
1732                return null;
1733            }
1734        }
1735    
1736        public static List<CCPSlotMention> getMultipleSlotMentionsByName(CCPTextAnnotation ccpTextAnnotation, String slotMentionName) {
1737            return getMultipleSlotMentionsByName(ccpTextAnnotation.getClassMention(), slotMentionName);
1738        }
1739    
1740        public static List<CCPSlotMention> getMultipleSlotMentionsByName(CCPClassMention ccpClassMention, String slotMentionName) {
1741            List<CCPSlotMention> returnSlotMentions = new ArrayList<CCPSlotMention>();
1742            FSArray slotMentionsArray = ccpClassMention.getSlotMentions();
1743            if (slotMentionsArray != null) {
1744                FeatureStructure[] slotMentions = slotMentionsArray.toArray();
1745                for (FeatureStructure fs : slotMentions) {
1746                    CCPSlotMention ccpSlotMention = (CCPSlotMention) fs;
1747                    if (ccpSlotMention.getMentionName().equals(slotMentionName)) {
1748                        returnSlotMentions.add(ccpSlotMention);
1749                    }
1750                }
1751                return returnSlotMentions;
1752            } else {
1753                return null;
1754            }
1755        }
1756    
1757        public static String getFirstSlotValue(CCPNonComplexSlotMention ccpNonComplexSlotMention) {
1758            String[] slotValues = ccpNonComplexSlotMention.getSlotValues().toArray();
1759            return slotValues[0];
1760        }
1761    
1762        public static void addSlotValue(CCPClassMention ccpClassMention, String slotMentionName, String slotValue) throws CASException {
1763            CCPNonComplexSlotMention ccpNonComplexSlotMention = (CCPNonComplexSlotMention) UIMA_Util.getSlotMentionByName(ccpClassMention,
1764                    slotMentionName);
1765            if (ccpNonComplexSlotMention == null) { // then we need to create a new
1766                // CCPNonComplexSlotMention
1767                ccpNonComplexSlotMention = new CCPNonComplexSlotMention(ccpClassMention.getCAS().getJCas());
1768                ccpNonComplexSlotMention.setMentionName(slotMentionName);
1769                addSlotMention(ccpClassMention, ccpNonComplexSlotMention);
1770            }
1771            // add the slotvalue to the StringArray
1772            addToStringArray(ccpNonComplexSlotMention, slotValue);
1773        }
1774    
1775        private static void addToStringArray(CCPNonComplexSlotMention ccpNonComplexSlotMention, String slotValue) throws CASException {
1776            StringArray slotValues = ccpNonComplexSlotMention.getSlotValues();
1777            if (slotValues == null) {
1778                slotValues = new StringArray(ccpNonComplexSlotMention.getCAS().getJCas(), 1);
1779                slotValues.set(0, slotValue);
1780                ccpNonComplexSlotMention.setSlotValues(slotValues);
1781            } else {
1782                String[] previousValues = slotValues.toArray();
1783                int index = 0;
1784                slotValues = new StringArray(ccpNonComplexSlotMention.getCAS().getJCas(), previousValues.length + 1);
1785                for (String prevValue : previousValues) {
1786                    slotValues.set(index++, prevValue);
1787                }
1788                slotValues.set(index, slotValue);
1789            }
1790            ccpNonComplexSlotMention.setSlotValues(slotValues);
1791        }
1792    
1793        private static void addSlotMention(CCPClassMention ccpClassMention, CCPSlotMention ccpSlotMention) throws CASException {
1794            FSArray slotMentions = ccpClassMention.getSlotMentions();
1795            if (slotMentions == null) {
1796                slotMentions = new FSArray(ccpClassMention.getCAS().getJCas(), 1);
1797                slotMentions.set(0, ccpSlotMention);
1798            } else {
1799                FeatureStructure[] featureStructures = slotMentions.toArray();
1800                int index = 0;
1801                slotMentions = new FSArray(ccpClassMention.getCAS().getJCas(), featureStructures.length + 1);
1802                for (FeatureStructure fs : featureStructures) {
1803                    slotMentions.set(index++, fs);
1804                }
1805                slotMentions.set(index, ccpSlotMention);
1806            }
1807            ccpClassMention.setSlotMentions(slotMentions);
1808        }
1809    
1810        /**
1811         * Create a string that includes all of the spans within this mention. This includes recursively pulling out slot spans as well.
1812         * 
1813         * @param mention
1814         *            The mention to process
1815         * @return A string summarizing all the text spans included in this mention
1816         */
1817        private static String getCCPClassMentionString(CCPClassMention mention) {
1818            // Create text spans for the base annotation and all the slots
1819            ArrayList<TextSpan> textSpans = new ArrayList<TextSpan>();
1820            addClassMentionSpans(textSpans, mention);
1821            // Assemble the sorted spans into a single string
1822            StringBuffer sb = new StringBuffer();
1823            TextSpan previous = null;
1824            for (TextSpan span : textSpans) {
1825                if (previous == null) {
1826                    sb.append(span.text);
1827                } else if ((span.start - previous.end) <= 1) {
1828                    sb.append(" ");
1829                    sb.append(span.text);
1830                } else {
1831                    sb.append(" ... ");
1832                    sb.append(span.text);
1833                }
1834                previous = span;
1835            }
1836            return sb.toString();
1837        }
1838    
1839        /**
1840         * Add all of the text spans that take part in a mention into a sorted list of text spans.
1841         * 
1842         * @param textSpans
1843         *            The sorted list of text spans
1844         * @param mention
1845         *            The mention from which spans are to be extracted
1846         * @return The sorted list of text spans
1847         */
1848        private static ArrayList<TextSpan> addClassMentionSpans(ArrayList<TextSpan> textSpans, CCPClassMention mention) {
1849            // Grab the original document text from the JCas
1850            String data = null;
1851            try {
1852                data = mention.getCAS().getJCas().getDocumentText();
1853            } catch (CASException e) {
1854                return textSpans;
1855            }
1856            // Get the base annotation for this mention (it should point to a
1857            // CCPTextAnnotation)
1858            FSArray annotations = mention.getCcpTextAnnotations();
1859            if ((annotations == null) || (annotations.size() <= 0))
1860                return textSpans;
1861            Object jcasAnnotation = annotations.get(0);
1862            CCPTextAnnotation annotation = null;
1863            if (jcasAnnotation instanceof CCPTextAnnotation)
1864                annotation = (CCPTextAnnotation) jcasAnnotation;
1865            if (annotation == null)
1866                return textSpans;
1867            // Get all the spans from the base annotation
1868            FSArray spans = annotation.getSpans();
1869            if (spans == null) {
1870                sortIn(textSpans, new TextSpan(annotation.getStart(), annotation.getEnd(), data.substring(annotation.getStart(), annotation
1871                        .getEnd())));
1872            } else {
1873                for (int i = 0; i < spans.size(); i++) {
1874                    Object jcasSpan = spans.get(i);
1875                    CCPSpan span = null;
1876                    if (jcasSpan instanceof CCPSpan) {
1877                        span = (CCPSpan) jcasSpan;
1878                    }
1879                    if (span != null) {
1880                        sortIn(textSpans, new TextSpan(span.getSpanStart(), span.getSpanEnd(), data.substring(span.getSpanStart(), span
1881                                .getSpanEnd())));
1882                    }
1883                }
1884            }
1885            // Now sort in all the spans from the mention slots
1886            FSArray slots = mention.getSlotMentions();
1887            if (slots != null) {
1888                for (int i = 0; i < slots.size(); i++) {
1889                    Object jcasSlotMention = slots.get(i);
1890                    CCPComplexSlotMention slot = null;
1891                    if (jcasSlotMention instanceof CCPComplexSlotMention)
1892                        slot = (CCPComplexSlotMention) jcasSlotMention;
1893                    if (slot != null) {
1894                        FSArray slotClassMentions = slot.getClassMentions();
1895                        for (int j = 0; j < slotClassMentions.size(); j++) {
1896                            Object slotClassMention = slotClassMentions.get(j);
1897                            CCPClassMention classMention = null;
1898                            if (slotClassMention instanceof CCPClassMention)
1899                                classMention = (CCPClassMention) slotClassMention;
1900                            if (classMention != null) {
1901                                addClassMentionSpans(textSpans, classMention);
1902                            }
1903                        }
1904                    }
1905                }
1906            }
1907            // Done
1908            return textSpans;
1909        }
1910    
1911        /**
1912         * Sort a new text span into a list of text spans.
1913         * 
1914         * @param spans
1915         *            The sorted list of spans
1916         * @param span
1917         *            The new span to add
1918         * @return The sorted list of spans
1919         */
1920        private static ArrayList<TextSpan> sortIn(ArrayList<TextSpan> spans, TextSpan span) {
1921            if (spans.isEmpty()) {
1922                spans.add(span);
1923            } else {
1924                boolean found = false;
1925                for (int i = 0; i < spans.size(); i++) {
1926                    if (span.end <= spans.get(i).start) {
1927                        spans.add(i, span);
1928                        found = true;
1929                        break;
1930                    }
1931                }
1932                if (!found)
1933                    spans.add(span);
1934            }
1935            return spans;
1936        }
1937    
1938        public static Iterator<CCPTextAnnotation> getAnnotationsWithinSpan(Span span, JCas jcas) {
1939            return getAnnotationsWithinSpan(span, jcas, CCPTextAnnotation.type);
1940        }
1941    
1942        /**
1943         * return all annotations within the exact same span as the input span
1944         * 
1945         * @param span
1946         * @param jcas
1947         * @return
1948         */
1949        public static Iterator getAnnotationsWithinSpan(Span span, JCas jcas, int annotationType) {
1950    
1951            if (DEBUG) {
1952                System.out.println("Looking for annotations within " + span.getSpanStart() + " -- " + span.getSpanEnd());
1953            }
1954            /* Get a reference to the CAS and the CAS ConstraintFactory */
1955            CAS cas = jcas.getCas();
1956            ConstraintFactory cf = cas.getConstraintFactory();
1957    
1958            /* Constraints are built from tests and feature-paths */
1959            /* First build the tests */
1960            FSIntConstraint gtEqToSpanStart = cf.createIntConstraint();
1961            gtEqToSpanStart.geq(span.getSpanStart());
1962    
1963            FSIntConstraint ltEqToSpanEnd = cf.createIntConstraint();
1964            ltEqToSpanEnd.leq(span.getSpanEnd());
1965    
1966            /* Get handles to the features, use the type system */
1967            TypeSystem ts = cas.getTypeSystem();
1968    
1969            Feature beginFeature = ts.getFeatureByFullName(CAS.FEATURE_FULL_NAME_BEGIN);
1970            Feature endFeature = ts.getFeatureByFullName(CAS.FEATURE_FULL_NAME_END);
1971    
1972            /* Create a feature path for each feature */
1973            FeaturePath pathToBeginValue = cas.createFeaturePath();
1974            pathToBeginValue.addFeature(beginFeature);
1975            FeaturePath pathToEndValue = cas.createFeaturePath();
1976            pathToEndValue.addFeature(endFeature);
1977    
1978            /*
1979             * Connect the tests to the feature paths (s = the span of the trigger annotation, c = the span of the phrases and tokens to compare)
1980             */
1981    
1982            /**
1983             * is it within the span
1984             * 
1985             * <pre>
1986             *                        ccccc
1987             *                       ssssssss
1988             * </pre>
1989             */
1990            FSMatchConstraint testBegin = cf.embedConstraint(pathToBeginValue, gtEqToSpanStart);
1991            FSMatchConstraint testEnd = cf.embedConstraint(pathToEndValue, ltEqToSpanEnd);
1992    
1993            /* AND the tests for each of the three cases, then OR the AND'ed tests together */
1994            FSMatchConstraint testBoth = cf.and(testBegin, testEnd);
1995    
1996            /* Create a filtered iterator that uses this constraint */
1997            Iterator<CCPTextAnnotation> iter = (Iterator<CCPTextAnnotation>) cas.createFilteredIterator(jcas.getJFSIndexRepository()
1998                    .getAnnotationIndex(annotationType).iterator(), testBoth);
1999    
2000            return iter;
2001    
2002        }
2003    
2004        public static Iterator<CCPTextAnnotation> getOverlappingAnnotations(CCPTextAnnotation ccpTA, JCas jcas, int annotType) {
2005            Span span = null;
2006            try {
2007                span = new Span(ccpTA.getBegin(), ccpTA.getEnd());
2008            } catch (InvalidSpanException e) {
2009                e.printStackTrace();
2010            }
2011    
2012            return getAnnotationsEncompassingSpan(span, jcas, annotType);
2013    
2014        }
2015    
2016        public static Iterator<CCPTextAnnotation> getAnnotationsEncompassingSpan(Span span, JCas jcas) {
2017            return getAnnotationsEncompassingSpan(span, jcas, CCPTextAnnotation.type);
2018        }
2019    
2020        /**
2021         * return all annotations that contain the input span
2022         * 
2023         * @param span
2024         * @param jcas
2025         * @return
2026         */
2027        public static Iterator<CCPTextAnnotation> getAnnotationsEncompassingSpan(Span span, JCas jcas, int annotType) {
2028    
2029            if (DEBUG) {
2030                System.out.println("Looking for annotations overlapping " + span.getSpanStart() + " -- " + span.getSpanEnd());
2031            }
2032            /* Get a reference to the CAS and the CAS ConstraintFactory */
2033            CAS cas = jcas.getCas();
2034            ConstraintFactory cf = cas.getConstraintFactory();
2035    
2036            /* Constraints are built from tests and feature-paths */
2037            /* First build the tests */
2038            FSIntConstraint ltEqToSpanStart = cf.createIntConstraint();
2039            ltEqToSpanStart.leq(span.getSpanStart());
2040            FSIntConstraint gtSpanStart = cf.createIntConstraint();
2041            gtSpanStart.gt(span.getSpanStart());
2042            FSIntConstraint gtEqToSpanStart = cf.createIntConstraint();
2043            gtEqToSpanStart.geq(span.getSpanStart());
2044    
2045            FSIntConstraint gtEqToSpanEnd = cf.createIntConstraint();
2046            gtEqToSpanEnd.geq(span.getSpanEnd());
2047            FSIntConstraint ltSpanEnd = cf.createIntConstraint();
2048            ltSpanEnd.lt(span.getSpanEnd());
2049            FSIntConstraint ltEqToSpanEnd = cf.createIntConstraint();
2050            ltEqToSpanEnd.leq(span.getSpanEnd());
2051    
2052            /* Get handles to the features, use the type system */
2053            TypeSystem ts = cas.getTypeSystem();
2054    
2055            Feature beginFeature = ts.getFeatureByFullName(CAS.FEATURE_FULL_NAME_BEGIN);
2056            Feature endFeature = ts.getFeatureByFullName(CAS.FEATURE_FULL_NAME_END);
2057    
2058            /* Create a feature path for each feature */
2059            FeaturePath pathToBeginValue = cas.createFeaturePath();
2060            pathToBeginValue.addFeature(beginFeature);
2061            FeaturePath pathToEndValue = cas.createFeaturePath();
2062            pathToEndValue.addFeature(endFeature);
2063    
2064            /*
2065             * Connect the tests to the feature paths (s = the span of the trigger annotation, c = the span of the phrases and tokens to compare)
2066             */
2067    
2068            /**
2069             * does it overlap the left
2070             * 
2071             * <pre>
2072             *                   cccccccc
2073             *                       ssssssss
2074             * </pre>
2075             */
2076            FSMatchConstraint testBegin1 = cf.embedConstraint(pathToBeginValue, ltEqToSpanStart);
2077            FSMatchConstraint testEnd1 = cf.embedConstraint(pathToEndValue, gtSpanStart);
2078            /**
2079             * does it overlap the right
2080             * 
2081             * <pre>
2082             *                     ccccccccc 
2083             *                  sssssss
2084             * </pre>
2085             */
2086            FSMatchConstraint testBegin2 = cf.embedConstraint(pathToBeginValue, ltSpanEnd);
2087            FSMatchConstraint testEnd2 = cf.embedConstraint(pathToEndValue, gtEqToSpanEnd);
2088            /**
2089             * is it completely in the middle
2090             * 
2091             * <pre>
2092             *                   cccc 
2093             *                 sssssssssss
2094             * </pre>
2095             */
2096            FSMatchConstraint testBegin3 = cf.embedConstraint(pathToBeginValue, gtEqToSpanStart);
2097            FSMatchConstraint testEnd3 = cf.embedConstraint(pathToEndValue, ltEqToSpanEnd);
2098    
2099            /* AND the tests for each of the three cases, then OR the AND'ed tests together */
2100            FSMatchConstraint testBoth1 = cf.and(testBegin1, testEnd1);
2101            FSMatchConstraint testBoth2 = cf.and(testBegin2, testEnd2);
2102            FSMatchConstraint testBoth3 = cf.and(testBegin3, testEnd3);
2103    
2104            FSMatchConstraint testBoth12 = cf.or(testBoth1, testBoth2);
2105            FSMatchConstraint testBoth123 = cf.or(testBoth12, testBoth3);
2106    
2107            /* Create a filtered iterator that uses this constraint */
2108            Iterator<CCPTextAnnotation> iter = (Iterator<CCPTextAnnotation>) cas.createFilteredIterator(jcas.getJFSIndexRepository()
2109                    .getAnnotationIndex(annotType).iterator(), testBoth123);
2110    
2111            return iter;
2112    
2113        }
2114    
2115        // public static Iterator<CCPTextAnnotation> getAnnotationsNotOfType(int ccpAnnotationType, List<String> excludeCCPAnnotationTypes, JCas
2116        // jcas) {
2117        // if (DEBUG) {
2118        // System.out.println("Looking for annotations of type " + ccpAnnotationType + " but not of type... ");
2119        // }
2120        // /* Get a reference to the CAS and the CAS ConstraintFactory */
2121        // CAS cas = jcas.getCas();
2122        // ConstraintFactory cf = cas.getConstraintFactory();
2123        //
2124        // /* Get handles to the features, use the type system */
2125        // TypeSystem ts = cas.getTypeSystem();
2126        //        
2127        // /* Constraints are built from tests and feature-paths */
2128        // /* First build the tests */
2129        // for (String typeStr : excludeCCPAnnotationTypes) {
2130        // Type type = ts.getType(typeStr);
2131        // FSTypeConstraint notType = cf.createTypeConstraint();
2132        // notType.add(type);
2133        // }
2134        //
2135        // Feature beginFeature = ts.getFeatureByFullName(TCAS.FEATURE_FULL_NAME_BEGIN);
2136        // Feature endFeature = ts.getFeatureByFullName(TCAS.FEATURE_FULL_NAME_END);
2137        //
2138        // /* Create a feature path for each feature */
2139        // FeaturePath pathToBeginValue = cas.createFeaturePath();
2140        // pathToBeginValue.addFeature(beginFeature);
2141        // FeaturePath pathToEndValue = cas.createFeaturePath();
2142        // pathToEndValue.addFeature(endFeature);
2143        //
2144        // /*
2145        // * Connect the tests to the feature paths (s = the span of the trigger annotation, c = the span of the phrases and tokens to
2146        // * compare)
2147        // */
2148        //
2149        // /**
2150        // * does it overlap the left
2151        // *
2152        // * <pre>
2153        // * cccccccc
2154        // * ssssssss
2155        // * </pre>
2156        // */
2157        // FSMatchConstraint testBegin1 = cf.embedConstraint(pathToBeginValue, ltEqToSpanStart);
2158        // FSMatchConstraint testEnd1 = cf.embedConstraint(pathToEndValue, gtSpanStart);
2159        // /**
2160        // * does it overlap the right
2161        // *
2162        // * <pre>
2163        // * ccccccccc
2164        // * sssssss
2165        // * </pre>
2166        // */
2167        // FSMatchConstraint testBegin2 = cf.embedConstraint(pathToBeginValue, ltSpanEnd);
2168        // FSMatchConstraint testEnd2 = cf.embedConstraint(pathToEndValue, gtEqToSpanEnd);
2169        // /**
2170        // * is it completely in the middle
2171        // *
2172        // * <pre>
2173        // * cccc
2174        // * sssssssssss
2175        // * </pre>
2176        // */
2177        // FSMatchConstraint testBegin3 = cf.embedConstraint(pathToBeginValue, gtEqToSpanStart);
2178        // FSMatchConstraint testEnd3 = cf.embedConstraint(pathToEndValue, ltEqToSpanEnd);
2179        //
2180        // /* AND the tests for each of the three cases, then OR the AND'ed tests together */
2181        // FSMatchConstraint testBoth1 = cf.and(testBegin1, testEnd1);
2182        // FSMatchConstraint testBoth2 = cf.and(testBegin2, testEnd2);
2183        // FSMatchConstraint testBoth3 = cf.and(testBegin3, testEnd3);
2184        //
2185        // FSMatchConstraint testBoth12 = cf.or(testBoth1, testBoth2);
2186        // FSMatchConstraint testBoth123 = cf.or(testBoth12, testBoth3);
2187        //
2188        // /* Create a filtered iterator that uses this constraint */
2189        // Iterator<CCPTextAnnotation> iter = (Iterator<CCPTextAnnotation>) cas.createFilteredIterator(jcas.getJFSIndexRepository()
2190        // .getAnnotationIndex(CCPTextAnnotation.type).iterator(), testBoth123);
2191        //
2192        // return iter;
2193        //        
2194        //        
2195        //        
2196        //        
2197        //        
2198        // }
2199    
2200        /**
2201         * return annotations in between spans
2202         * 
2203         * @param span
2204         * @param jcas
2205         * @return
2206         */
2207        public static Iterator<CCPTextAnnotation> getAnnotationsInBetweenSpans(Span upstreamSpan, Span downstreamSpan, JCas jcas) {
2208    
2209            if (DEBUG) {
2210                System.out.println("Looking for annotations in between " + upstreamSpan.getSpanEnd() + " -- " + downstreamSpan.getSpanStart());
2211            }
2212    
2213            Span betweenSpan = null;
2214            try {
2215                betweenSpan = new Span(upstreamSpan.getSpanEnd(), downstreamSpan.getSpanStart());
2216            } catch (InvalidSpanException e) {
2217                error("Invalid span detected.. could be because of split span. These are not handled. [" + upstreamSpan.getSpanEnd() + ".." + downstreamSpan.getSpanStart() + "]");
2218                return null;
2219                // e.printStackTrace();
2220            }
2221    
2222            if (betweenSpan != null) {
2223                return getAnnotationsWithinSpan(betweenSpan, jcas);
2224            } else {
2225                return null;
2226            }
2227    
2228        }
2229    
2230        /**
2231         * return all annotations that start with the input startIndex
2232         * 
2233         * @param startIndex
2234         * @param jcas
2235         * @return
2236         */
2237        public static Iterator<CCPTextAnnotation> getAnnotationsWithSameStart(int startIndex, JCas jcas) {
2238    
2239            if (DEBUG) {
2240                System.out.println("Looking for annotations starting at " + startIndex);
2241            }
2242    
2243            /* Get a reference to the CAS and the CAS ConstraintFactory */
2244            CAS cas = jcas.getCas();
2245            ConstraintFactory cf = cas.getConstraintFactory();
2246    
2247            /* Constraints are built from tests and feature-paths */
2248            /* First build the tests */
2249            FSIntConstraint eqToSpanStart = cf.createIntConstraint();
2250            eqToSpanStart.eq(startIndex);
2251    
2252            /* Get handles to the features, use the type system */
2253            TypeSystem ts = cas.getTypeSystem();
2254    
2255            Feature beginFeature = ts.getFeatureByFullName(CAS.FEATURE_FULL_NAME_BEGIN);
2256    
2257            /* Create a feature path for each feature */
2258            FeaturePath pathToBeginValue = cas.createFeaturePath();
2259            pathToBeginValue.addFeature(beginFeature);
2260    
2261            /*
2262             * Connect the tests to the feature paths (s = the span of the trigger annotation, c = the span of the phrases and tokens to compare)
2263             */
2264    
2265            /**
2266             * does it start at the same index
2267             * 
2268             * <pre>
2269             *                   cccccccc
2270             *                   ssssssss
2271             * </pre>
2272             */
2273            FSMatchConstraint testStart = cf.embedConstraint(pathToBeginValue, eqToSpanStart);
2274    
2275            /* Create a filtered iterator that uses this constraint */
2276            Iterator<CCPTextAnnotation> iter = (Iterator<CCPTextAnnotation>) cas.createFilteredIterator(jcas.getJFSIndexRepository()
2277                    .getAnnotationIndex(CCPTextAnnotation.type).iterator(), testStart);
2278    
2279            return iter;
2280    
2281        }
2282    
2283        public static Iterator<CCPTextAnnotation> getPrecedingAnnotations(int startIndex, int ccpAnnotationType, JCas jcas) {
2284    
2285            if (DEBUG) {
2286                System.out.println("Looking for annotations ending before  " + startIndex);
2287            }
2288    
2289            /* Get a reference to the CAS and the CAS ConstraintFactory */
2290            CAS cas = jcas.getCas();
2291            ConstraintFactory cf = cas.getConstraintFactory();
2292    
2293            /* Constraints are built from tests and feature-paths */
2294            /* First build the tests */
2295            FSIntConstraint ltSpanStart = cf.createIntConstraint();
2296            ltSpanStart.lt(startIndex);
2297    
2298            /* Get handles to the features, use the type system */
2299            TypeSystem ts = cas.getTypeSystem();
2300    
2301            Feature endFeature = ts.getFeatureByFullName(CAS.FEATURE_FULL_NAME_END);
2302    
2303            /* Create a feature path for each feature */
2304            FeaturePath pathToEndValue = cas.createFeaturePath();
2305            pathToEndValue.addFeature(endFeature);
2306    
2307            /*
2308             * Connect the tests to the feature paths (s = the span of the trigger annotation, c = the span of the phrases and tokens to compare)
2309             */
2310    
2311            /**
2312             * does it start at the same index
2313             * 
2314             * <pre>
2315             *                   cccccccc
2316             *                   ssssssss
2317             * </pre>
2318             */
2319            FSMatchConstraint testStart = cf.embedConstraint(pathToEndValue, ltSpanStart);
2320    
2321            /* Create a filtered iterator that uses this constraint */
2322            Iterator<CCPTextAnnotation> iter = (Iterator<CCPTextAnnotation>) cas.createFilteredIterator(jcas.getJFSIndexRepository()
2323                    .getAnnotationIndex(ccpAnnotationType).iterator(), testStart);
2324    
2325            return iter;
2326    
2327        }
2328    
2329        /**
2330         * this method resets the span for an annotation.. this includes the Span FSArray
2331         * 
2332         * @param ccpTA
2333         * @param spanStart
2334         * @param spanEnd
2335         */
2336        public static void setCCPTextAnnotationSpan(CCPTextAnnotation ccpTA, int spanStart, int spanEnd) throws CASException {
2337            JCas jcas = ccpTA.getCAS().getJCas();
2338    
2339            /* Initialize a new CCPSpan */
2340            CCPSpan ccpSpan = new CCPSpan(jcas);
2341            ccpSpan.setSpanStart(spanStart);
2342            ccpSpan.setSpanEnd(spanEnd);
2343    
2344            /* Add the span to the span array */
2345            FSArray ccpSpans = new FSArray(jcas, 1);
2346            ccpSpans.set(0, ccpSpan);
2347    
2348            /* Update the text annotation with the new span information */
2349            ccpTA.setSpans(ccpSpans);
2350            ccpTA.setBegin(spanStart);
2351            ccpTA.setEnd(spanEnd);
2352    
2353            /* update class mention to point to this text annotation */
2354            CCPClassMention ccpCM = ccpTA.getClassMention();
2355            if (ccpCM != null) {
2356                FSArray ccpTAs = new FSArray(jcas, 1);
2357                ccpTAs.set(0, ccpTA);
2358                ccpCM.setCcpTextAnnotations(ccpTAs);
2359            }
2360    
2361        }
2362    
2363        /**
2364         * sets the class mention for the CCPTextAnnotation, and also links the CCPTextAnnotation with the CCPClassMention
2365         * 
2366         * @param ccpTA
2367         * @param ccpCM
2368         * @throws CASException
2369         */
2370        public static void setCCPPClassMentionForCCPTextAnnotation(CCPTextAnnotation ccpTA, CCPClassMention ccpCM) throws CASException {
2371            ccpTA.setClassMention(ccpCM);
2372            addCCPTextAnnotationToCCPClassMention(ccpTA, ccpCM);
2373        }
2374    
2375        public static void addCCPTextAnnotationToCCPClassMention(CCPTextAnnotation ccpTA, CCPClassMention ccpCM) throws CASException {
2376            JCas jcas = ccpTA.getCAS().getJCas();
2377    
2378            FSArray taArray = ccpCM.getCcpTextAnnotations();
2379            if (taArray == null) {
2380                taArray = new FSArray(jcas, 1);
2381                taArray.set(0, ccpTA);
2382                ccpCM.setCcpTextAnnotations(taArray);
2383            } else {
2384                /* this class mention already has some text annotations associated with it, or it has an initialized, but empty, FSArray */
2385                FSArray newTAarray = new FSArray(jcas, taArray.size() + 1);
2386                for (int i = 0; i < taArray.size(); i++) {
2387                    newTAarray.set(i, taArray.get(i));
2388                }
2389                newTAarray.set(newTAarray.size() - 1, ccpTA);
2390                ccpCM.setCcpTextAnnotations(newTAarray);
2391            }
2392            
2393        }
2394    
2395        /**
2396         * This method provides a sanity check when constructing CCPTextAnnotations, and in particular the class mention structure from scratch. Slots are
2397         * checked for appropriate filler, and error messages are raised if an invalid slot filler is discovered. True is returned if the
2398         * CCPTextAnnotation has a valid mention structure, false otherwise.
2399         * 
2400         * @param ccpTA
2401         * @return
2402         */
2403        public static boolean validateCCPTextAnnotation(CCPTextAnnotation ccpTA) {
2404            boolean isValid =  validateCCPClassMention(ccpTA.getClassMention());
2405            UIMA_Util uimaUtil = new UIMA_Util();
2406            error("Invalid class mention structure detected in CCPTextAnnotation: " + uimaUtil.getSingleLineRepresentationOfCCPTextAnnotation(ccpTA, false));
2407            return isValid;
2408        }
2409    
2410        /**
2411         * This method checks each slot mention associated with the input class mention for valid slot fillers. True is returned if the CCPClassMention
2412         * has a valid mention structure, false otherwise.
2413         * 
2414         * @param ccpTA
2415         * @return
2416         */
2417        public static boolean validateCCPClassMention(CCPClassMention ccpCM) {
2418            boolean isValid = true;
2419    
2420            FSArray shouldBeSlotMentions = ccpCM.getSlotMentions();
2421            for (int i = 0; i < shouldBeSlotMentions.size(); i++) {
2422                Object shouldBeSM = shouldBeSlotMentions.get(i);
2423                if (shouldBeSM instanceof CCPSlotMention) {
2424                    return validateCCPSlotMention((CCPSlotMention) shouldBeSM);
2425                } else {
2426                    error("Invalid mention structure detected. Unexpected object found in FSArray holding CCPSlotMentions for the CCPClassMention: \""
2427                            + ccpCM.getMentionName() + "\" -- " + shouldBeSM.getClass().getName());
2428                    return false;
2429                }
2430            }
2431    
2432            FSArray shouldBeTextAnnotations = ccpCM.getCcpTextAnnotations();
2433            for (int i = 0; i < shouldBeTextAnnotations.size(); i++) {
2434                Object shouldBeTA = shouldBeTextAnnotations.get(i);
2435                if (!(shouldBeTA instanceof CCPTextAnnotation)) {
2436                    error("Invalid mention structure detected. Expected CCPTextAnnotation linked to CCPClassMention: " + ccpCM.getMentionName()
2437                            + " but instead found a: " + shouldBeTA.getClass().getName());
2438                    return false;
2439                }
2440            }
2441    
2442            return isValid;
2443    
2444        }
2445    
2446        /**
2447         * This method determines the type of slot mention inputted, and calls either validateCCPComplexSlotMention() or
2448         * validateCCPNonComplexSlotMention()
2449         * 
2450         * @param ccpSM
2451         * @return
2452         */
2453        public static boolean validateCCPSlotMention(CCPSlotMention ccpSM) {
2454            if (ccpSM instanceof CCPComplexSlotMention) {
2455                return validateCCPComplexSlotMention((CCPComplexSlotMention) ccpSM);
2456            } else if (ccpSM instanceof CCPNonComplexSlotMention) {
2457                return validateCCPNonComplexSlotMention((CCPNonComplexSlotMention) ccpSM);
2458            } else {
2459                error("The superclass CCPSlotMention was found to occupy a slot. Only subclasses of CCPSlotMention are allowed.");
2460                return false;
2461            }
2462        }
2463    
2464        /**
2465         * This method checks for valid slot fillers of the input CCPComplexSlotMention, i.e. slot fillers must be valid CCPClassMentions. True is
2466         * returned if the CCPClassMention has a valid mention structure, false otherwise.
2467         * 
2468         * @param ccpTA
2469         * @return
2470         */
2471        public static boolean validateCCPComplexSlotMention(CCPComplexSlotMention ccpCSM) {
2472            boolean isValid = true;
2473    
2474            FSArray slotValues = ccpCSM.getClassMentions();
2475            for (int i = 0; i < slotValues.size(); i++) {
2476                Object shouldBeAClassMention = slotValues.get(i);
2477                if (shouldBeAClassMention instanceof CCPClassMention) {
2478                    isValid = isValid && validateCCPClassMention((CCPClassMention) shouldBeAClassMention);
2479                } else {
2480                    error("Invalid mention structure discovered. Instead of a CCPClassMention, this slot filler for this CCPComplexSlotMention is a: "
2481                            + shouldBeAClassMention.getClass().getName());
2482                    return false;
2483                }
2484            }
2485    
2486            return isValid;
2487        }
2488    
2489        /**
2490         * This method checks for valid slot fillers of the input CCPNonComplexSlotMention, i.e. slot fillers must be Strings. This method is not
2491         * necessary in truth because the StringArray object of each CCPNonComplexSlotMention will force the slot values to be strings, but is included
2492         * for completeness.
2493         * 
2494         * @param ccpTA
2495         * @return
2496         */
2497        public static boolean validateCCPNonComplexSlotMention(CCPNonComplexSlotMention ccpNCSM) {
2498            /*
2499             * the CCPNonComplexSlotMention is forced to hold Strings, so its mention structure will always be valid. This method is included only for
2500             * completeness.
2501             */
2502            return true;
2503        }
2504    
2505        private static void error(String message) {
2506            System.err.println("ERROR -- UIMA_Util: " + message);
2507        }
2508    
2509    }
2510    
2511    /**
2512     * A local class to hold a span of text along with its document start and end position.
2513     * 
2514     * @author Jim Firby
2515     */
2516    class TextSpan {
2517    
2518        int start = 0;
2519    
2520        int end = 0;
2521    
2522        String text = null;
2523    
2524        TextSpan(int start, int end, String text) {
2525            this.start = start;
2526            this.end = end;
2527            this.text = text;
2528        }
2529    
2530    }
```
